# Ultrasound‐Recharged Sub‐Nanometer Palladium Catalysts for on‐Demand and Self‐Terminating Bioorthogonal Prodrug Activation in Cancer Therapy

**DOI:** 10.1002/advs.76338

**Published:** 2026-07-01

**Authors:** Daqing Xia, Lei Liu, Shuang Jin, Guangxu Fang, Chang Yu, Yunyun Wu, Lunli Xiang, Hongrui Zhu, Zhenqiang Wang, Jixi Zhang

**Affiliations:** ^1^ Key Laboratory of Biorheological Science and Technology College of Bioengineering Ministry of Education Chongqing University Chongqing China; ^2^ Department of Pharmacy The Second Affiliated Hospital Army Medical University Chongqing China

**Keywords:** bioorthogonal chemistry, closed‐Loop catalysis, immunogenic cell death, piezocatalysis

## Abstract

Bioorthogonal catalysis holds great promise for precision cancer therapy, yet its clinical translation is critically hindered by the lack of reliable control over catalytic activity. Uncontrolled “always‐on” catalysis or irreversible activation prevents timely termination, making it difficult to confine prodrug activation within a safe therapeutic window. To address this challenge, we developed an ultrasound‐recharging‐regulated closed‐loop bioorthogonal system integrating on‐demand activation, enhanced catalytic performance, and self‐termination, enabling precise in vivo prodrug activation. Mechanistically, pyroelectric polarization of BaTiO_3_ induces deposition of sub‐nanometer Pd clusters whose catalysis is initially silenced by rapid oxidative passivation. Upon ultrasound stimulation, the piezoelectric BaTiO_3_ core generates a built‐in electric field that reduces surface Pd^2+^ to catalytically active Pd^0^, thereby activating bioorthogonal reactions while promoting charge separation and reactive oxygen species generation. Simultaneously, piezoelectric screening charges accelerate interfacial electron transfer, significantly enhancing catalytic kinetics. Crucially, rapid repassivation after ultrasound cessation leads to complete catalytic deactivation, establishing a closed bioorthogonal cycle following a single stimulus. In vitro and in vivo studies demonstrate that the activated species induce immunogenic cell death while avoiding side effects from persistent catalysis. This work presents a safe, controllable, and efficient paradigm for bioorthogonal catalytic therapy, advancing its clinical translation.

## Graphical abstract

Safe and precise bioorthogonal therapy of tumors was achieved by an ultrasound‐responsive heterostructured nanoreactor with on‐demand catalytic activity. Under piezoelectric catalysis, the system undergoes reversible Pd^2^^+^/Pd^0^ redox cycling, concurrently enabling spatiotemporally controlled prodrug activation and self‐terminating catalytic dose confinement at the tumor site. This work paves a new avenue for designing closed‐loop catalytic systems toward precision cancer therapy.

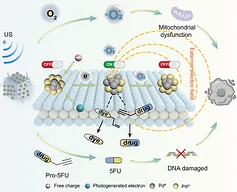

## 1 Introduction

Bioorthogonal chemistry, a cornerstone of modern chemical biology, enables highly selective and biocompatible chemical transformations within complex biological environments. This capability has driven its rapid adoption across a broad range of biomedical applications, including drug delivery [1, 2, 3, 4], bioimaging [5, 6, 7, 8, 9], and tumor therapy [9, 10, 11, 12]. Among its diverse toolkit, transition metal‐mediated bioorthogonal catalysis [13, 14, 15, 16], particularly palladium (Pd)‐based systems [17, 18, 19], enables the in situ synthesis of therapeutic agents, offering a potent strategy to minimize off‐target effects and enhance therapeutic efficacy. However, the clinical translation of Pd‐catalyzed prodrug activation is critically hindered by the inability to precisely control the activation dose at the target site. Conventional systems often operate in an uncontrolled, “always‐on” manner, lacking the spatiotemporal precision needed to confine activity within the optimal therapeutic window and avoid systemic toxicity.

To address these challenges, a variety of control strategies have been explored; however, none have achieved the integrated regulation required for safe and precise in vivo therapy. External stimulus–gated systems, such as UV‐ or near‐infrared–triggered catalysis [20, 21], are intrinsically constrained by limited tissue penetration and insufficient activation depth, particularly in deep‐seated tumors. Tumor microenvironment‐responsive biological logic‐based designs, including DNA‐gated [22] and logic‐gated nanogenerator [23], improve target specificity but typically operate as irreversible, single‐use platforms that lack autonomous deactivation, leaving residual catalytic activity after initial activation. In parallel, material‐based approaches aimed at enhancing catalytic kinetics, such as those employing liquid metals [24] or black phosphorus [25], often maintain a persistent “always‐on” state. This further increases the risk of uncontrolled prodrug activation. A recent study has reported a piezoelectric‐assisted bioorthogonal catalytic system; however, it primarily relies on stimulus‐enhanced activation without reversible catalytic switching or autonomous termination [26]. Collectively, these limitations underscore that an ideal bioorthogonal catalytic system for clinical translation must simultaneously fulfill three stringent requirements: (i) remote activation with deep‐tissue penetration, (ii) high catalytic efficiency, and (iii) an intrinsic self‐termination mechanism that enables spatiotemporally precise dose control within the therapeutic window. Therefore, overcoming these challenges demands a fundamentally different strategy that moves beyond incremental optimization of individual components, namely one in which all three requirements are intrinsically coupled through a unified physicochemical mechanism.

From this perspective, external fields capable of deep‐tissue penetration emerge as a natural entry point. Among these modalities, ultrasound uniquely combines clinical translatability, deep tissue penetration, and high spatiotemporal addressability, making it particularly well suited for dynamically regulating in vivo catalytic processes. Importantly, the multiple accessible oxidation states of palladium provide a chemical basis for constructing reversible catalytic switches, while electron‐rich environments can effectively accelerate reaction kinetics. Consequently, a platform that integrates ultrasound responsiveness with reversible Pd valence regulation and efficient electron donation offers a logical route toward achieving on‐demand activation, kinetic enhancement, and autonomous deactivation within a single system. Notably, efficient and controllable bioorthogonal catalysis typically relies on nanostructures with high surface area and tunable active sites [27, 28], making sub‐nanoscale Pd catalysts key candidate materials. Their prominent interfacial effects and electronic properties [29, 30, 31] not only exhibit high catalytic activity in physiological environments but also provide an ideal platform for constructing multifunctional rapid‐response systems. However, significant challenges remain in synthesizing sub‐nanometer Pd clusters and precisely regulating their surface electronic states in complex physiological environments to achieve efficient, reversible catalytic functions and on‐demand therapy.

Ferroelectric barium titanate (BaTiO_3_) offers a unique opportunity to overcome these challenges. As a multifunctional ferroelectric material, BaTiO_3_ can generate electron‐hole pairs through modulation of its spontaneous polarization under thermal (pyroelectric) or mechanical (piezoelectric) stimulation [32, 33, 34, 35, 36, 37, 38, 39, 40, 41, 42]. Under periodic temperature variations, the pyroelectric effect enriches electrons at the reduction end of the material, enabling selective reduction and immobilization of noble metals, thereby providing a feasible route for in situ deposition of sub‐nanometer Pd clusters [43, 44]. Simultaneously, the piezoelectric effect allows BaTiO_3_ to function as an “ultrasound‐rechargeable battery,” converting mechanical vibrations into redox potentials capable of dynamically regulating transition metal valence states [45, 46, 47, 48]. Moreover, the external electric field generated by piezoelectric screening charges has been shown to significantly accelerate click chemistry reactions [49], suggesting an additional pathway for enhancing bioorthogonal reaction kinetics. Together, these properties position BaTiO_3_ as a uniquely suitable platform for constructing an ultrasound‐regulated, reversible, and kinetically enhanced bioorthogonal catalytic system.

In this study, an ultrasound‐controlled smart closed‐loop bioorthogonal catalysis system was designed to address the challenge of achieving spatiotemporally precise control over prodrug activation dosage in traditional palladium‐based bioorthogonal catalysis (Scheme 1). Specifically, ultrasmall barium titanate nanocrystals (BA) were synthesized via a two‐phase solvothermal method to possess both pyroelectric and piezoelectric properties. Subsequently, their surface was modified with dihydrocaffeic acid ligands to obtain hydrophilic BT‐DHCA (BD) nanoparticles. Under thermal cycling conditions, Pd sub‐nanometer clusters were in situ reduced and deposited on the BD surface to construct tunable catalytic sites. Notably, superoxide radicals generated during the pyroelectric process were exploited to rapidly passivate the Pd catalytic interface, thereby rendering the system initially in a catalytically “silent” state (OFF). Under periodic ultrasound excitation, the induced cavitation effect was utilized to trigger the piezocatalytic process: the alternating built‐in electric field was employed to promote sonoluminescence‐induced electron–hole pair separation, so as to significantly enhance charge carrier separation efficiency and thereby drive massive generation of reactive oxygen species (ROS). Concurrently, electrons were efficiently harnessed to reduce surface Pd^2+^ to Pd^0^, in order to successfully “start” the catalytic activity. In addition, the external electric field formed by piezo‐shielding charges was applied to accelerate the dealkynylation process of the 5‐fluorouracil (5FU) prodrug, thereby enabling highly efficient in situ generation of the chemotherapeutic agent. Crucially, an intrinsic self‐termination mechanism was incorporated into the system: after ultrasound cessation, ROS were mediated to re‐passivate Pd^0^, so that the bioorthogonal catalytic system could be rapidly returned to the inactive (OFF) state and immediate self‐termination could be achieved. Thus, excessive activation and off‐target release of the prodrug in non‐target areas were prevented to ensure precision and safety in treatment. Finally, in vitro and in vivo experiments were conducted to demonstrate that ROS‐mediated oxidative stress and 5FU‐induced antimetabolite therapy can synergistically trigger robust immunogenic cell death (ICD).

**SCHEME 1:**
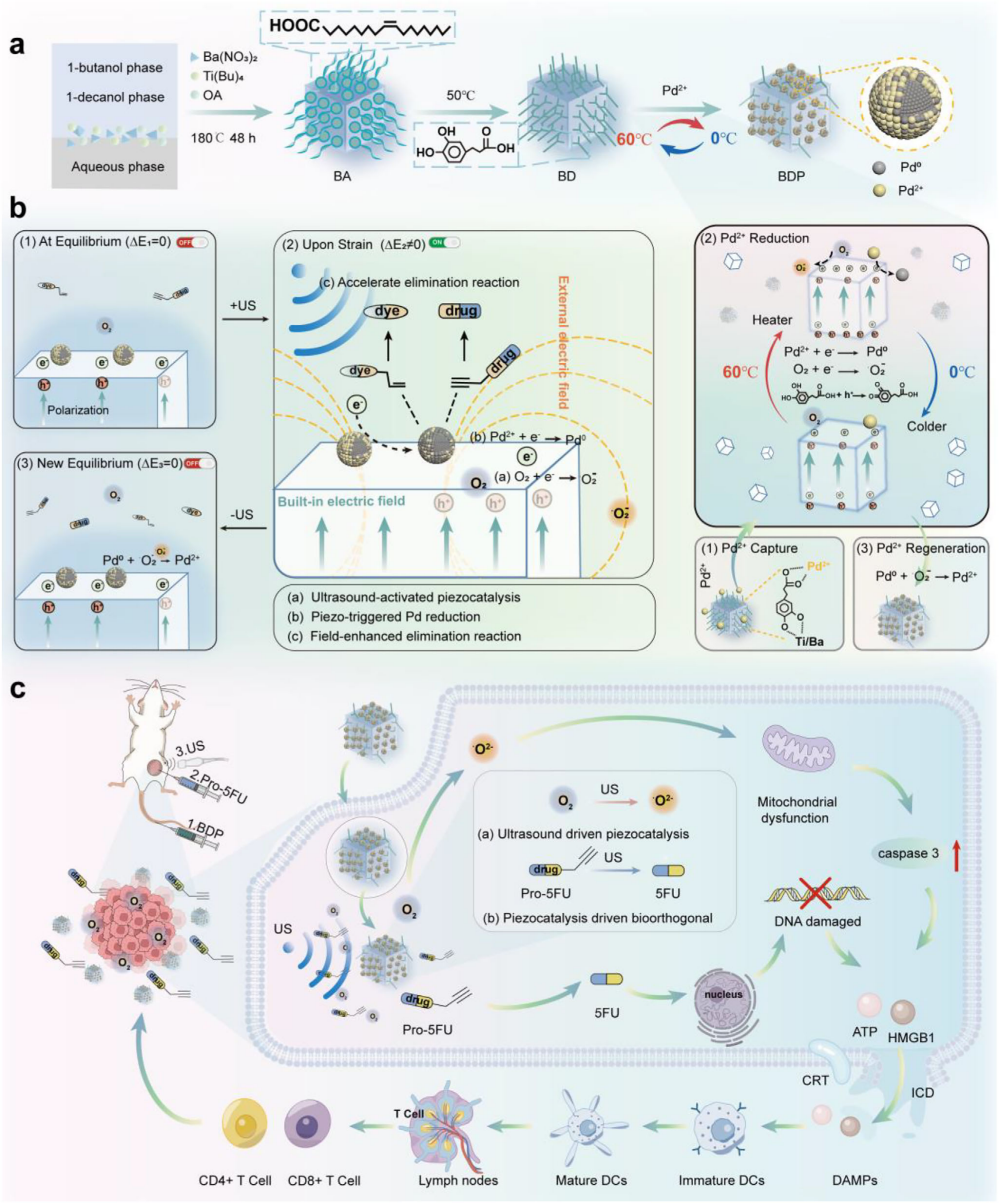
Schematic illustration of the design and application of the switchable bioorthogonal catalysis. (a) A schematic diagram of selective deposition of Pd on ultrasmall barium titanate surfaces via pyroelectric catalysis and metal chelation. (b) The working mechanism of piezoelectric activation and oxidative termination of the bioorthogonal catalysis. (c) The underlying mechanism of BDP for synergistic tumor immune‐therapy. BDP was activated and accelerated by the built‐in electric field and piezoelectric field for catalyzing bioorthogonal uncaging reaction and producing ROS, leading to an ICD‐mediated immunotherapy.

## 2 Results and Discussion

### 2.1 Synthesis and Characterization of BaTiO_3_‐Pd

A facile strategy was developed for the reductive deposition of sub‐nanometric Pd composites on ultrasmall ferroelectric barium titanate (BaTiO_3_) nanoparticles. Initially, hydrophobic oleic acid‐stabilized BaTiO_3_ (BA) was synthesized via a hydrothermal method [34, 50]. Ligand exchange was then performed using dihydrocaffeic acid (DHCA) [51], which leverages the strong catechol coordination to convert BA into hydrophilic BaTiO_3_ (BD) with abundant surface carboxyl groups (Figure 1a). Capitalizing on the carboxyl‐mediated metal‐chelation ability and the intrinsic pyroelectricity of BaTiO_3_, Pd was in situ deposited on the reductive poles of BD under 0°C–60°C thermal cycling, forming piezoelectric BaTiO_3_‐supported Pd composite particles (BDP). Transmission electron microscopy (TEM) images revealed that the hydrothermally synthesized BaTiO_3_ nanoparticles were highly uniform, with an average size of approximately 10 nm (Figure 1b; Figures S1 and S2). After DHCA modification, the resulting BD nanoparticles exhibited excellent aqueous dispersibility (Figure 1c; Figure S3), as corroborated by macroscopic phase transfer observations (Figure S4). X‐ray diffraction (XRD) and Raman spectroscopy (Figure S5a,b) confirmed the typical ferroelectric tetragonal phase of the as‐synthesized BaTiO_3_, implying good piezoelectricity, further supported by piezoelectric force microscopy (PFM) measurements (Figure S6). Moreover, electrochemical measurements during 0°C–60°C thermal cycling (Figure S7) detected notable pyroelectric currents (Figure S8), further indicating strong pyroelectric performance. These results demonstrate that BD can promote redox reactions in response to ambient temperature fluctuations, enabling reductive deposition of noble metals. Simultaneously, the abundant carboxyl groups from DHCA facilitate the effective capture of Pd^2+^ ions from solution (Figure 1d,e; Figure S9). Accordingly, the Pd reduction process during thermal cycling was systematically investigated.

**FIGURE 1:**
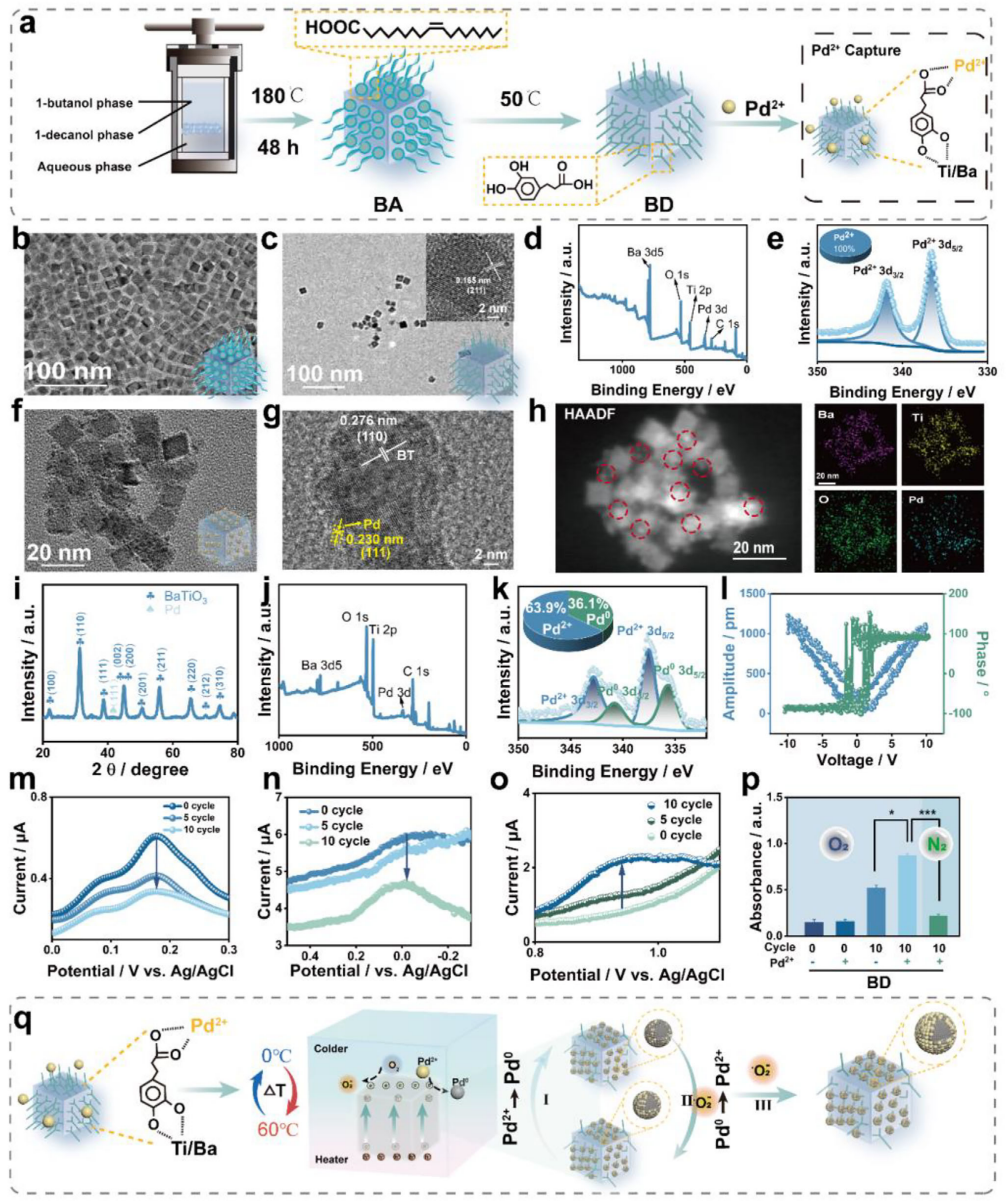
Synthesis and characterization of BA, BD, and BDP. (a) Schematic diagram for the preparation of BDP nanocatalysts. (b) TEM image of BaTiO_3_ nanocubes coated with oleic acid. (c) TEM image of BaTiO_3_ nanocubes coated with DHCA; the inset shows an HRTEM image of BD NPs. (d) XPS survey scan spectrum of the composite particles formed after co‐incubation of BD with Pd^2+^ for 12 h. (e) High‐resolution XPS spectrum and peak deconvolution of Pd 3d. (f) TEM image of BDP. (g) HRTEM image of BDP NPs. (h) Dark‐field image and elemental maps of BDP. (i) XRD pattern of BDP nanocubes. (j) XPS survey spectrum of BDP NPs. (k) High‐resolution XPS spectrum and peak deconvolution of Pd 3d; inset shows the proportion of Pd^2+^ and Pd^0^ in the BDP. (l) The corresponding piezo‐response force microscopy (PFM) phase and amplitude hysteresis loops for BDP NPs. DPV measurements of oxidation of surface‐bound DHCA (m), reduction of Pd^2+^ in solution (n), and oxidation of deposited Pd^0^ on particles (o). (p) Absorption intensity at 560 nm of NBT in BD under O_2_ (left) or N_2_ (right) atmosphere in the presence or absence of Pd^2+^ or upon 10 cycles of thermal shock. (q) Schematic illustration of the principle for pyroelectric deposition of BDP composite particles.

Monitoring the reaction system by UV–Vis spectroscopy revealed a gradual increase in absorbance and solution darkening with successive thermal cycles (Figure S10), while no significant change occurred at constant temperatures (0°C or 60°C) (Figures S11–S13), indicating the temperature‐cycling‐dependent nature of the Pd deposition process. TEM analysis further revealed the formation of sub‐nanometric particles on the BaTiO_3_ surface (Figure 1f). High‐resolution imaging and high‐angle annular dark‐field scanning TEM (HAADF‐STEM) elemental mapping confirmed that these particles were Pd‐rich, with the signal intensity increasing alongside cycle number (Figure 1g,h; Figures S14 and S15). The XRD pattern (Figure 1i) revealed characteristic diffraction peaks corresponding to the Pd phase. XPS survey and high‐resolution Pd 3d spectra (Figure 1j,k) revealed that Pd existed in both metallic (Pd^0^) and oxidized (Pd^2+^) states, with a high Pd^2+^ proportion of 63.9%. Piezoresponse force microscopy measurements indicated an enhanced piezoelectric response for the BDP composite (Figure 1l).

The high fraction of Pd^2+^ prompted a deeper investigation into the redox mechanism during thermal cycling. Differential pulse voltammetry (DPV) monitoring [52, 53] revealed the gradual oxidation of surface catechol groups (Figure 1m and Figure S16), a decrease in aqueous Pd^2+^ concentration (Figure 1n), and a concomitant increase in surface Pd° content (Figure 1o) upon cycling. This suggests that the catechol groups in DHCA act as hole scavengers, while the carboxyl‐anchored Pd^2+^ ions serve as nucleation sites, being reduced to Pd^0^ by electrons generated via the pyroelectric effect. As Pd^0^ is highly susceptible to oxidation, we hypothesized it could be re‐oxidized to PdO by reactive oxygen species (e.g., superoxide anion, ·O_2_^−^) generated during subsequent piezoelectric catalysis [54], leading to the coexistence of Pd^0^ and Pd^2+^ in BDP. To validate this, we examined piezoelectric‐driven O_2_^−^ generation under different atmospheres. Significant ·O_2_^−^ production was detected in air, which can oxidize Pd^0^. This is identified as the primary reason for the persistent high surface Pd^2+^ ratio after ultrasonication ceases (and the electron supply terminates) (Figure 1p). Under N_2_ atmosphere, TEM and XPS results (Figures S17 and S18) indicate that after 10 thermal cycles, Pd remains deposited on BD, and the Pd° content reaches as high as 73.1%. These results further validate that ·O_2_^−^ generated in air oxidizes Pd^0^ to PdO. These results conclusively demonstrate the successful fabrication of a surface‐passivated piezoelectric Pd‐based bioorthogonal catalyst (Figure 1q).

### 2.2 Ultrasound‐Responsive ROS Generation and Bioorthogonal Catalytic Performance

A systematic study was conducted to evaluate the ability of BDP to generate ROS via piezoelectric catalysis and its efficiency in initiating bioorthogonal reactions (Figure 2a). Electron spin resonance (ESR) spectroscopy using 5,5‐dimethyl‐1‐pyrroline N‐oxide (DMPO) as the spin trap revealed a characteristic six‐line signal for superoxide anion radicals (O_2_^−^), while no hydroxyl radical (·OH) signal was observed (Figure 2b,c), indicating specific ·O_2_^−^ production by BDP under ultrasound (US). The piezoelectric catalytic performance of BDP was quantitatively assessed by monitoring total ROS production with 2’,7’‐dichlorodihydrofluorescein (DCFH). As shown in Figure 2d, the fluorescence intensity at 525 nm in the BDP group increased gradually with ultrasound (US) time and was significantly higher than that of the blank and BD groups (Figure 2e), indicating that the heterostructure in BDP effectively enhances piezoelectric catalytic ROS generation. Further investigation revealed the relationship between ROS generation and the number of thermal cycles. As shown in Figure S19, ROS generation peaked after eight cycles. The diminished catalytic activity is likely due to excess Pd functioning as charge recombination centers, which promote electron‐hole recombination and reduce the efficiency of the catalytic process. Subsequent experiments will use BDP prepared through 8 thermal cycles as the experimental group. Identification of radical types using specific probes showed no detectable ·OH production by BDP (Figure 2f,g), likely due to the scavenging effect of surface catechol groups. In contrast, the absorbance of nitroblue tetrazolium (NBT) at 560 nm increased with US time (Figure 2h,i), and the response of the BDP group was significantly stronger than that of the control groups, consistent with the ESR results and confirming specific ·O_2_^−^ generation.

**FIGURE 2:**
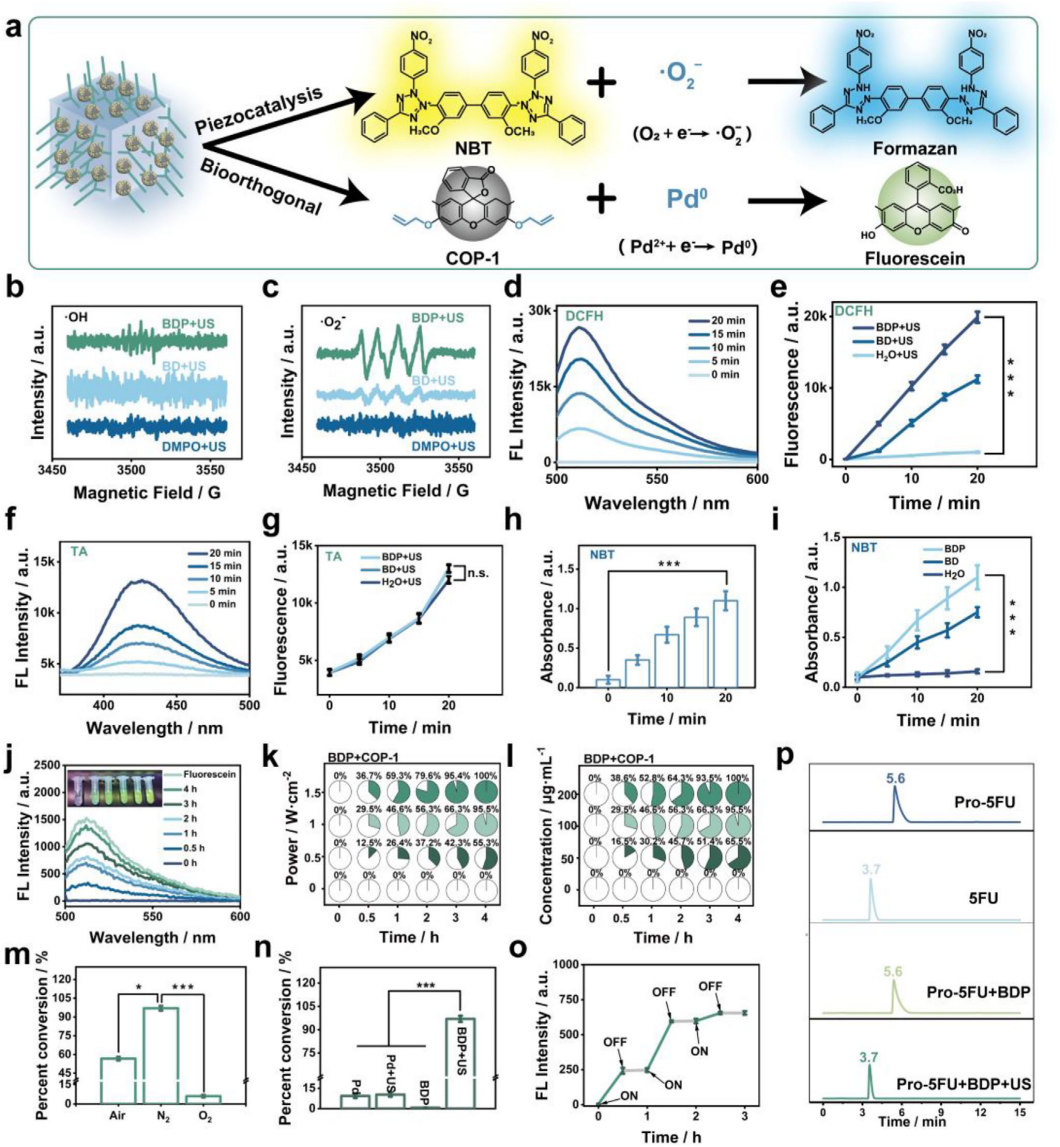
Characterizations of catalytic efficacy. (a) Schematic illustration of the detection methodology for piezocatalysis and bioorthogonal reaction. ESR spectra of ·O_2_^−^ (b) and ·OH (c) trapped by DMPO. (d) Time‐dependent fluorescence spectrum changes of DCFH in the presence of BDP under US irradiation. (e) Fluorescence change kinetics of DCFH with different treatments. Data were presented as mean ± SD (n = 3). (f) Time‐dependent fluorescence spectrum changes of TA in the presence of BDP under US irradiation. (g) Fluorescence change kinetics of TA with different treatments. Data were presented as mean ± SD (n = 3). (h) Time‐dependent change in the absorption intensity at λ = 560 nm of NBT in the presence of BDP under US irradiation. (i) Absorbance change kinetics of NBT with different treatments. Data were presented as mean ± SD (n = 3). (j) Time‐dependent fluorescence spectrum changes of COP‐1 in the presence of BDP under US irradiation; the inset shows photographs of the corresponding reaction solutions under ultraviolet light. (k) Time‐dependent fluorescence changes of COP‐1 in the presence of BDP (100 µg mL^−1^) in response to US irradiation at varying power densities. (l) Time‐dependent fluorescence changes of COP‐1 in response to BDP at varying concentrations (0, 50, 100, and 200 µg mL^−1^) under US irradiation (1.0 W cm^−2^). (m) Conversion efficiency of COP‐1 by BDP under different atmospheres (N_2_, Air, O_2)_ after a 4‐h reaction. Data were presented as mean ± SD (n = 3). (n) Conversion efficiency of COP‐1 by different groups (Pd‐PVP, BDP) with and without ultrasound under a N_2_ atmosphere after 4 h of reaction. Data were presented as mean ± SD (n = 3). (o) COP‐1 conversion by BDP under cycled ultrasound (0.5 h on/off) in N_2_. Data were presented as mean ± SD (n = 3). (p) HPLC quantification of time‐dependent prodrug cleavage by various experimental groups over 4 h. Significance was calculated using one‐way ANOVA and Tukey's multiple comparisons test. ^*^ *p* < 0.05, ^**^ *p* < 0.01, ^***^ *p* < 0.001, and n.s. represents no significant difference.

The US‐driven bioorthogonal reaction was evaluated using the COP‐1 fluorescent probe, which detects carbon monoxide (CO) generation via the Pd^0^‐mediated Tsuji‐Trost reaction [55, 56]. Under a N_2_ atmosphere, nearly complete conversion of the precursor probe was achieved within 4 h of US treatment (1.0 MHz, 1.0 W cm^−2^) using 100 µg mL^−1^ BDP (Figure 2j). The influence of US power and particle concentration was systematically examined. As shown in Figure 2k,l, the conversion rate per unit time increased significantly with increasing US power or BDP concentration. The effect of atmosphere was further evaluated over 4 h (Figure 2m), revealing near‐complete conversion under N_2_, approximately 50% conversion in air, and less than 10% under O_2_, indicating strong suppression of the piezocatalytic bioorthogonal reaction by oxygen. To highlight the contribution of piezoelectric catalysis, PVP‐modified Pd nanoparticles (Figures S20 and S21) were used as a control. As shown in Figure 2n, the conventional Pd catalyst achieved only about 10% conversion in 4 h, and US did not significantly enhance the reaction rate. In contrast, BDP enabled nearly complete conversion within the same period, demonstrating its superior catalytic performance. Inspired by this efficient acceleration, fluorescence intensity was monitored over time under intermittent ultrasound (ON‐OFF cycling) stimulation (Figure 2o). The fluorescence signal increased in a stepwise manner upon ultrasound activation and remained stable during the OFF intervals. This dynamic response confirms that the bioorthogonal reaction in this system exhibits excellent ultrasound‐gated characteristics, enabling on‐demand “OFF‐ON‐OFF” switching control. The uncaging efficiency, conversion rate, catalytic turnover number, and passivation behavior of the piezocatalytic bioorthogonal reaction for the prodrug Pro‐5FU (Figure S22) were further investigated. At 37°C, the piezocatalyst and the prodrug Pro‐5FU were dispersed in PBS at final concentrations of 100 µg mL^−^^1^ and 500 µm, respectively. The activation of the prodrug was quantitatively analyzed by HPLC. As shown in Figure 2p and Figure S23a, under ultrasound, approximately 99.5% of the prodrug was converted after 4 h of sonication. In contrast, without ultrasound, the conversion rate was less than 1% within the same period, strongly demonstrating that piezocatalysis effectively promotes the Pd‐mediated bioorthogonal reaction. The activation and passivation behavior of this piezocatalytic bioorthogonal system was further examined by monitoring prodrug conversion under intermittent (on–off cycle) ultrasound stimulation (Figure S23b). Upon ultrasound activation, the system initiated the bioorthogonal reaction to activate the prodrug; once ultrasound ceased, the conversion immediately stopped, exhibiting rapid passivation. The prodrug conversion showed a stepwise pattern corresponding to the on–off cycles of ultrasound. Moreover, during a single 30‐min continuous ultrasound period, the calculated catalytic turnover number (TON) was approximately 2.56. Based on the experimental data, this study conclusively demonstrates that the BDP heterostructure serves as a highly efficient and ultrasound‐gated platform, capable of both selectively generating superoxide radicals and mediating piezocatalytic bioorthogonal reactions with exceptional spatiotemporal control.

### 2.3 Mechanistic Investigation of Piezocatalysis and Dynamic Pd State Modulation

The high piezocatalytic performance and intelligent switching behavior of the system motivated a deeper investigation into the underlying mechanism. Piezocatalytic mechanisms are generally described by two models: the screening charge effect and the energy band theory [57]. As BaTiO_3_ possesses both ferroelectric and semiconducting properties, we first determined the band gaps (Eg) of BD and BDP using UV–Vis diffuse reflectance spectroscopy (Figure 3a), which were calculated to be 3.05 and 1.94 eV, respectively. Mott–Schottky plots displayed positive slopes (Figure 3b), confirming their n‐type semiconductor characteristics, with flat‐band potentials of −1.0 and −1.35 V (vs. Ag/AgCl), where *E*_Ag/AgCl_ is ≈0.2 V at room temperature with pH = 7 (*E_f_* (vs NHE) = *E_f_*  + *E*_Ag/AgCl_ + 0.0591*pH). For n‐type semiconductors, the flat‐band potential is generally close to the conduction band (CB) position; therefore, the CB edges were estimated to be approximately −0.39 and −0.74 V (vs. NHE), respectively. Accordingly, the valence band (VB) positions were calculated using Eg = CB‐VB, yielding VB values of 2.66 V (BD) and 2.31 V (BDP). The narrowed band gap of BDP facilitates electronic excitation by sonoluminescence generated during ultrasound cavitation. Electrochemical impedance spectroscopy (EIS) indicated a lower charge transfer resistance for BDP (Figure 3c; Figure S24), suggesting more efficient interfacial electron transfer. Photoluminescence (PL) spectroscopy further verified a prolonged charge carrier lifetime in BDP (Figure 3d). A comparison of photocurrent and sonocurrent responses (Figure 3e,f) revealed that BD exhibited a rapid photocurrent response under light irradiation, but a delayed rise and slow decay under ultrasound stimulation. In contrast, BDP showed delayed responses under both stimuli: the delayed photocurrent can be attributed to pyroelectric effects, whereas the delayed sonocurrent is likely associated with suppressed charge recombination under the external electric field. In summary, the piezocatalytic mechanism of BaTiO_3_ is co‐regulated by its energy band structure and built‐in and external electric fields. As illustrated in Figure 3g, the heterostructure formed between Pd and BaTiO_3_ effectively narrows the bandgap, promoting excitation via cavitation‐induced sonoluminescence. Meanwhile, the intrinsic structural asymmetry of the material generates a built‐in electric field that induces band bending and facilitates the separation of photoinduced charge carriers. In addition, the external electric field effectively suppresses charge recombination, significantly extending the carrier lifetime. These effects act synergistically to enhance the efficiency of ROS generation via piezocatalysis.

**FIGURE 3:**
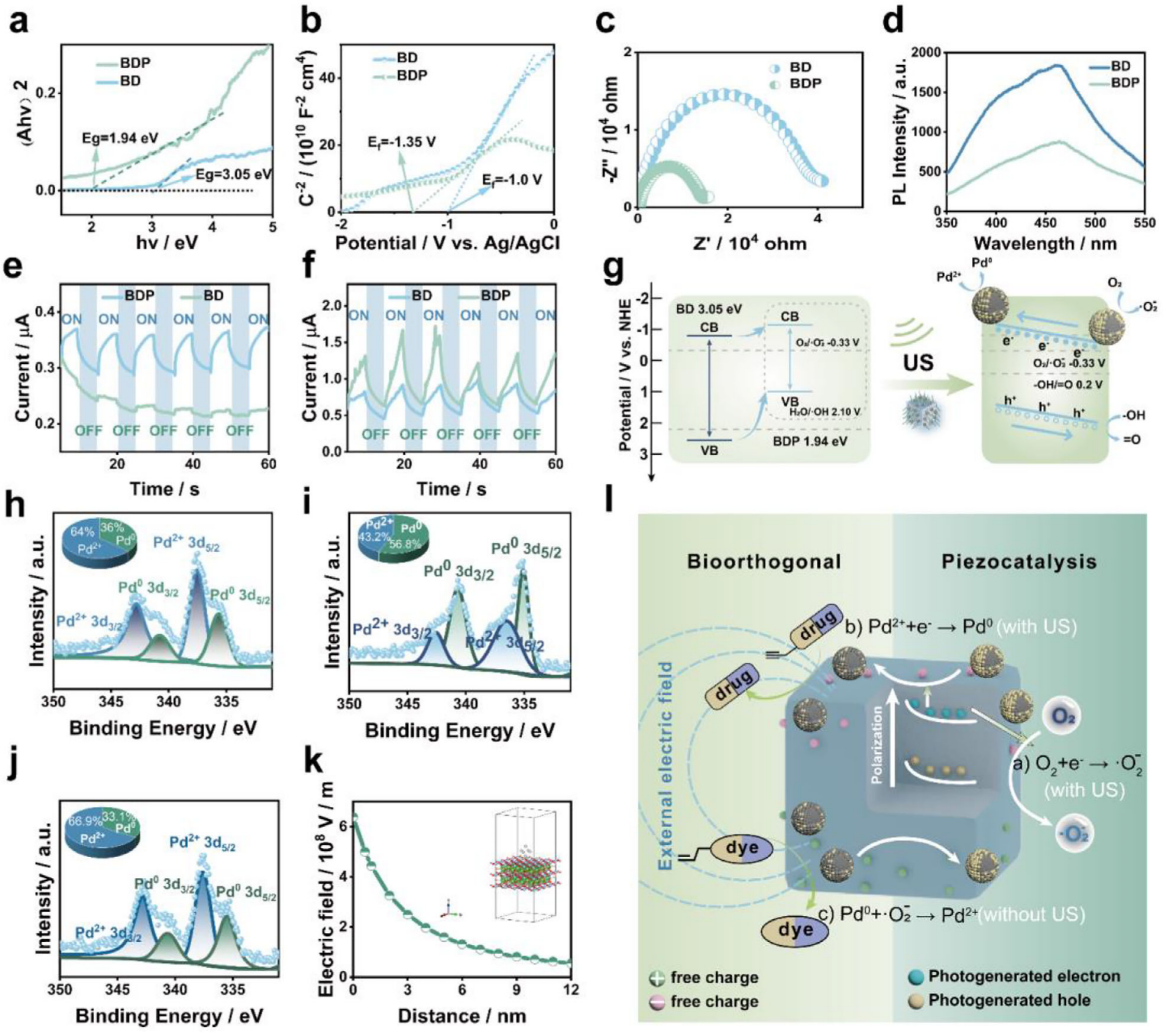
Analysis of catalysis mechanisms. Bandgap energies (a), Mott‐Schottky curves (b), related ElS Nyquist curves (c), and Photoluminescence (PL) spectroscopy (d) of BD and BDP. Photocurrent (e) and sonocurrent responses (f) of BD and BDP. (g) The schematic illustration of the band structure (vs NHE (normalized hydrogen electrode)). High‐resolution XPS spectra of Pd 3d of initial BDP (h) and BDP (i) after 4 h of US irradiation under N_2_ atmosphere, as well as BDP (j) after 4 h of US irradiation under O_2_ atmosphere. (k) Theoretical calculations of the reaction kinetics at the Pd interface. (l) The schematic illustration of the piezoelectric catalysis‐activated bioorthogonal uncaging reaction and ROS production.

The dynamic modulation of the Pd valence state is crucial for switching the bioorthogonal reaction between the ON and OFF states. XPS analysis revealed that pristine BDP exhibited a high proportion of Pd^2+^ (64%, Figure 3h), indicating that the surface is predominantly composed of Pd^2+^, corresponding to the OFF state of the bioorthogonal reaction in the absence of ultrasound. After 4 h of ultrasound treatment under an N_2_ atmosphere, the proportion of Pd^0^ increased significantly from 36% to 56.8% (Figure 3i), demonstrating that piezocatalytically generated electrons effectively reduce surface Pd^2+^ to Pd^0^, thereby activating the bioorthogonal reaction and switching the system to the ON state. In contrast, when ultrasound was applied in an O_2_ atmosphere, the Pd° content decreased slightly from 36% to 33.1% (Figure 3j), confirming that superoxide anions (·O_2_^−^) generated during piezocatalysis rapidly reoxidize Pd^0^ to Pd^2+^, thereby maintaining the OFF state. These results conclusively demonstrate that the BDP composite enables reversible modulation of the Pd valence state via the piezoelectric effect, allowing precise dynamic switching of the bioorthogonal reaction between ON and OFF states. Theoretical calculations further revealed (Figure 3k) that the strong external electric field generated during piezocatalysis significantly accelerates the reaction kinetics at the Pd interface, providing a mechanistic basis for the enhanced bioorthogonal reaction rate. Importantly, once ultrasound ceases, the piezoelectric electron supply terminates. In the presence of oxygen, superoxide anions rapidly reoxidize Pd^0^ to Pd^2+^, restoring the system to its original OFF state and enabling reversible catalytic deactivation (Figure 3l).

### 2.4 In Vitro and In Vivo Antitumor Efficacy of Piezocatalytic Bioorthogonal Therapy

Based on the demonstrated intelligent switchable bioorthogonal catalytic capability of BDP, we further evaluated its catalytic efficacy at the cellular level. First, the colloidal stability of BDP was investigated in different dispersion media. As shown in the inset of Figure S25a, BDP exhibited no visible sedimentation or agglomeration over 7 days in all tested media (Water, PBS, and DMEM). Hydrodynamic size measurements (Figure S25a, main panel) revealed a slight, gradual increase in the average diameter of BDP dispersed in water over time. Correspondingly, the autocorrelation functions in Figure S25b decayed progressively more slowly with increasing days. Nevertheless, all correlation functions decayed smoothly and completely to the baseline without any distortion, indicating that the observed increase in size is not due to partial aggregation. This behavior is most likely attributed to surface oxidation of Pd on the BDP particles: exposure to air leads to the formation of a PdO layer, which enhances surface hydrophilicity and results in a thicker hydration shell, thereby increasing the overall hydrodynamic size; yet the dispersion remains colloidally stable, suggesting a low risk of vascular occlusion. Then, the biocompatibility and cytotoxicity of BDP and Pro‐5FU were assessed. As shown in Figure 4a,b, after 24 h of incubation, 500 µg mL^−1^ BDP NPs and 500 µm Pro‐5FUL929 cell viability remained above 90% for both materials, indicating good biocompatibility. The optimal BDP and Pro‐5FU concentration for subsequent studies was determined by assessing cell viability under ultrasonication at varying doses. Under ultrasound irradiation, the viability of 4T1 cells decreased with increasing concentrations of Pro‐5FU and BDP in a concentration‐dependent manner. At 200 µg mL^−^^1^ BDP and 500 µm Pro‐5FU, the cell viability dropped to approximately 5% (Figure S26), which is superior to most previously reported Pd‐mediated bioorthogonal catalytic systems (see Table S1 for comparison). Given this excellent performance, a prodrug concentration of 500 µm was selected for subsequent cellular studies. Further investigation of prodrug activation at the cellular level was carried out using LC‐MS. Analysis of cell lysates subjected to varying ultrasound treatment times revealed that in the experimental group treated with ultrasound for 4 h, the mass spectra (Figure S27) clearly displayed peaks corresponding to 5FU and hydroxyacetone, indicating that the prodrug was activated via a dealkynylation pathway. HPLC results (Figure S28) demonstrated the activation efficiency of the prodrug at the cellular level, with the conversion rate increasing as the ultrasound treatment time was prolonged (Table S2). Under the employed experimental conditions (1.0 MHz, 1.0 W cm^−^^2^, 50% duty cycle, 5 min, 200 µg mL^−^^1^ BDP, 500 µm Pro‐5FU), a prodrug activation of 12.8% was achieved. Given the importance of cellular internalization for antitumor efficacy [58], the intracellular uptake of BDP nanoparticles was visualized using the membrane‐impermeable dye Cy7 (Figure S29a). To further elucidate the uptake mechanism, chemical inhibitors were employed in 4T1 cells (Figure S29b) [59, 60, 61, 62]. Chlorpromazine, an inhibitor of clathrin‐mediated endocytosis, drastically reduced cellular fluorescence to ∼4% of the untreated control level (29.1%), whereas nystatin (caveolae‐mediated) and EIPA (macropinocytosis) showed little effect. Thus, despite their negative surface charge, BDP nanoparticles enter cancer cells primarily via clathrin‐mediated endocytosis.

**FIGURE 4:**
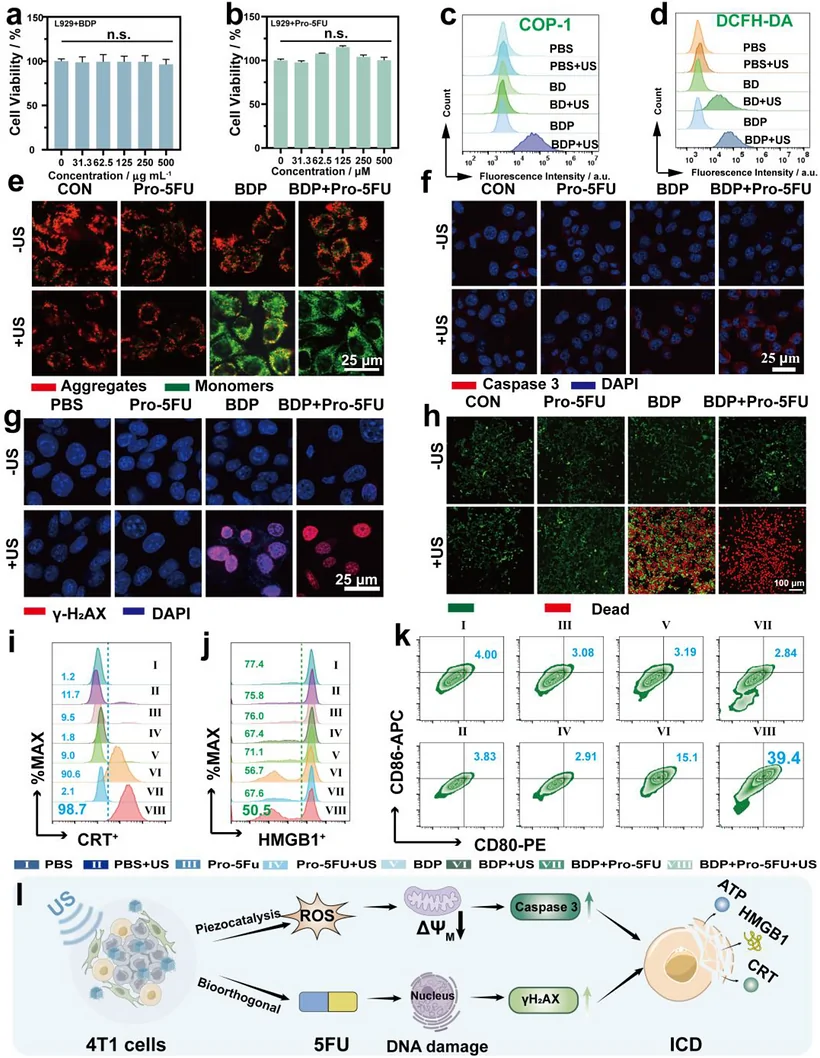
In vitro cellular assays to validate the synergistic immunotherapy performance of BDP. Cell viability of L929 cells treated with different concentrations of BDP (a) and Pro‐5FU (b). Data were presented as mean ± SD (n = 5). (c) Flow cytometry detection of bioorthogonal uncaging reactions activated by piezoelectric catalysis in 4T1 cells using COP‐1 as a probe. (d) Flow cytometric detection of ROS generation activated by piezoelectric catalysis in 4T1 cells using DCFH‐DA as a probe. (e) CLSM images of Mitochondrial membrane potential (Δψm) of 4T1 cells after different treatments using JC‐1 as a probe. (f) Caspase 3 assay of 4T1 cells after different treatments. (g) Fluorescence imaging of γ‐H_2_AX for analyzing DNA damage in 4T1 cells after different treatments. (h) Live and dead assay of 4T1 cells after different treatments. Flow cytometric detection of CRT (i) and HMGB1 (j) in 4T1 cells after different treatments. (k) Detection of DCs activation (marked by CD80 and CD86) in different treatment groups. (l) Schematic diagram of piezoelectric catalysis, activated ROS generation, and prodrug uncaging for ICD activation. Significance was calculated using one‐way ANOVA and Tukey's multiple comparisons test. ^*^ *p* < 0.05, ^**^ *p* < 0.01, ^***^ *p* < 0.001, n.s. represents no significant difference.

The ultrasound‐responsive piezocatalytic performance of BDP and its ability to initiate intracellular bioorthogonal reactions were subsequently investigated. The allyl ether‐based probe COP‐1, which emits green fluorescence upon Pd^0^‐mediated Tsuji‐Trost activation, was employed to verify the intracellular reaction [55, 56]. A strong fluorescence signal was observed in cells under ultrasound irradiation (Figure 4c and Figure S30), validating the successful initiation of the intracellular bioorthogonal reaction. Concurrently, ultrasound‐induced ROS generation was evaluated in 4T1 cells using DCFH‐DA staining. The BDP+US group exhibited the most pronounced increase in fluorescence (Figure 4d and Figure S31), which was attributed to the enhanced piezocatalytic activity of the BDP heterojunction. These collective results demonstrate that BDP possesses ultrasound‐responsive piezoelectric catalytic properties within cells and can effectively trigger intracellular bioorthogonal reactions. Substantial evidence indicates that elevated ROS levels disrupt mitochondrial membrane integrity and upregulate caspase‐3 expression, whereas the chemotherapeutic agent 5‐fluorouracil (5FU) induces DNA damage. The combined action of these two mechanisms promotes tumor cell death and activates immunogenic cell death (ICD). JC‐1 staining confirmed ultrasound‐induced mitochondrial dysfunction in the BDP+US and BDP+Pro‐5FU+US groups, as shown by a pronounced loss of ΔΨm indicated by the shift from red to green fluorescence (Figure 4e), which is attributed to piezocatalytically generated ROS. This finding was supported by robust caspase‐3 activation in the same treatment groups (Figure 4f), consistent with the induction of a mitochondrial‐dependent apoptotic pathway. Meanwhile, γ‐H_2_AX immunofluorescence revealed significant DNA damage in the BDP+Pro‐5FU+US group (Figure 4g), confirming genomic injury resulting from the ultrasound‐triggered bioorthogonal release of 5FU. The combination of DNA damage and mitochondrial dysfunction led to a near‐complete loss of cell viability in the BDP+Pro‐5FU+US group (Figure 4h; Figures S32 and S33), demonstrating the highest tumor cell lethality and underscoring the efficacy of the combined therapeutic strategy.

Flow cytometry analysis further revealed that BDP+Pro‐5FU+US treatment most effectively induced immunogenic cell death, as indicated by the coordinated upregulation of surface‐exposed calreticulin (CRT) and extracellular ATP release, together with HMGB1 release (Figure 4i,j; Figure S34). To evaluate DC maturation, a transwell co‐culture system was established with murine 4T1 cells in the upper chamber and immature BMDCs in the lower chamber. The 4T1 cells were treated with PBS, Pro‐5FU, BDP, or BDP+Pro‐5FU, with or without US. DCs were then collected and stained for CD11c, CD80, and CD86. As shown in Figure 4k, the BDP+Pro‐5FU+US group induced a marked increase in mature DCs (CD80^+^and CD86^+^ within CD11c^+^ population), with the proportion reaching 39.4%, which was approximately a 10‐fold increase relative to the control group. Together, these findings confirm that the combined action of piezocatalysis and bioorthogonal 5‐FU release elicits mitochondrial dysfunction, caspase‐3 activation, and DNA damage. These effects promote the release of damage‐associated molecular patterns (DAMPs), including HMGB1, CRT, and ATP, thereby enhancing dendritic cell maturation and antigen presentation (Figure 4l).

Motivated by the promising cellular performance of BDP, we further evaluated its therapeutic efficacy in a 4T1 tumor‐bearing mouse model. BDP exhibited an excellent biosafety profile in vivo, showing no obvious adverse effects on body weight (Figure S35) and minimal hemolysis (<8% at 2000 µg mL^−1^; Figure S36). No significant abnormalities were observed in blood routine (Figure S37), biochemical analyses (Figure S38), or histological examination of major organs, including the heart, liver, spleen, lung, and kidney (Figure S39). To determine the optimal timing for ultrasound application, the in vivo tumor accumulation of BDP‐loaded nanoparticles was first assessed using Cy7‐labeled BDP. As shown in Figure S40, the nanoparticles exhibited robust tumor accumulation within 12 h post‐injection, and this signal was sustained for up to 48 h. Ex vivo imaging further confirmed high tumor uptake, along with notable accumulation in the liver and kidneys. To quantitatively verify the peak accumulation time, we performed biodistribution analysis via ICP‐AES at 3, 6, 12, 24, and 48 h after intravenous injection (n = 3 per time point). The results revealed that the BDP content in tumors reached its maximum at 12 h (Figure S41), which was fully consistent with the fluorescence imaging data. Accordingly, 12 h post‐injection was selected as the time point for ultrasound treatment, ensuring that the prodrug administration coincided with maximal nanoparticle enrichment in the tumor. Furthermore, BDP was cleared primarily via the fecal route (40.63 ± 3.67% ID/g within 0–24 h), with a lower contribution from renal excretion (21.9 ± 5.24% ID/g; Figure S42).

Based on the favorable tumor targeting and biocompatibility of BDP, its tumoricidal efficacy was further investigated. Subsequently, 4T1 breast tumor‐bearing BALB/c mice were randomly assigned to seven groups (n = 5 per group): (I) Saline, (II) Pro‐5FU, (III) Pro‐5FU+US, (IV) BDP, (V) BDP+US, (VI) BDP+Pro‐5FU, and (VII) BDP+Pro‐5FU+US. After successful establishment of the breast tumor model (tumor volume ≈ 50 mm^3^), BDP was administered via tail vein injection. Pro‐5FU was subsequently injected intratumorally 12 h later. Ultrasound treatment was applied 30 min after Pro‐5FU administration (1.0 W cm^−2^, 50% duty cycle, 5 min per session) and repeated daily for 3 consecutive days. The body weight and tumor size of the mice were measured for 14 days (Figure 5a). Notably, neither the administration of Pro‐5FU alone, BDP alone, nor the combined administration of BDP and Pro‐5FU demonstrated a significant inhibitory effect on tumor development. In the ultrasound‐treated group, the BDP+US group exhibited a tumor growth inhibition rate of approximately 72%. In contrast, the BDP+Pro‐5FU+US group achieved a tumor growth inhibition rate of approximately 93.2%, which was significantly higher than that of the control group. Consistent with the above results, compared to the control group, the body weight of mice in the BDP+Pro‐5FU+US group showed a slight increase, while tumor volume and tumor size significantly decreased (Figure 5b–e).

**FIGURE 5:**
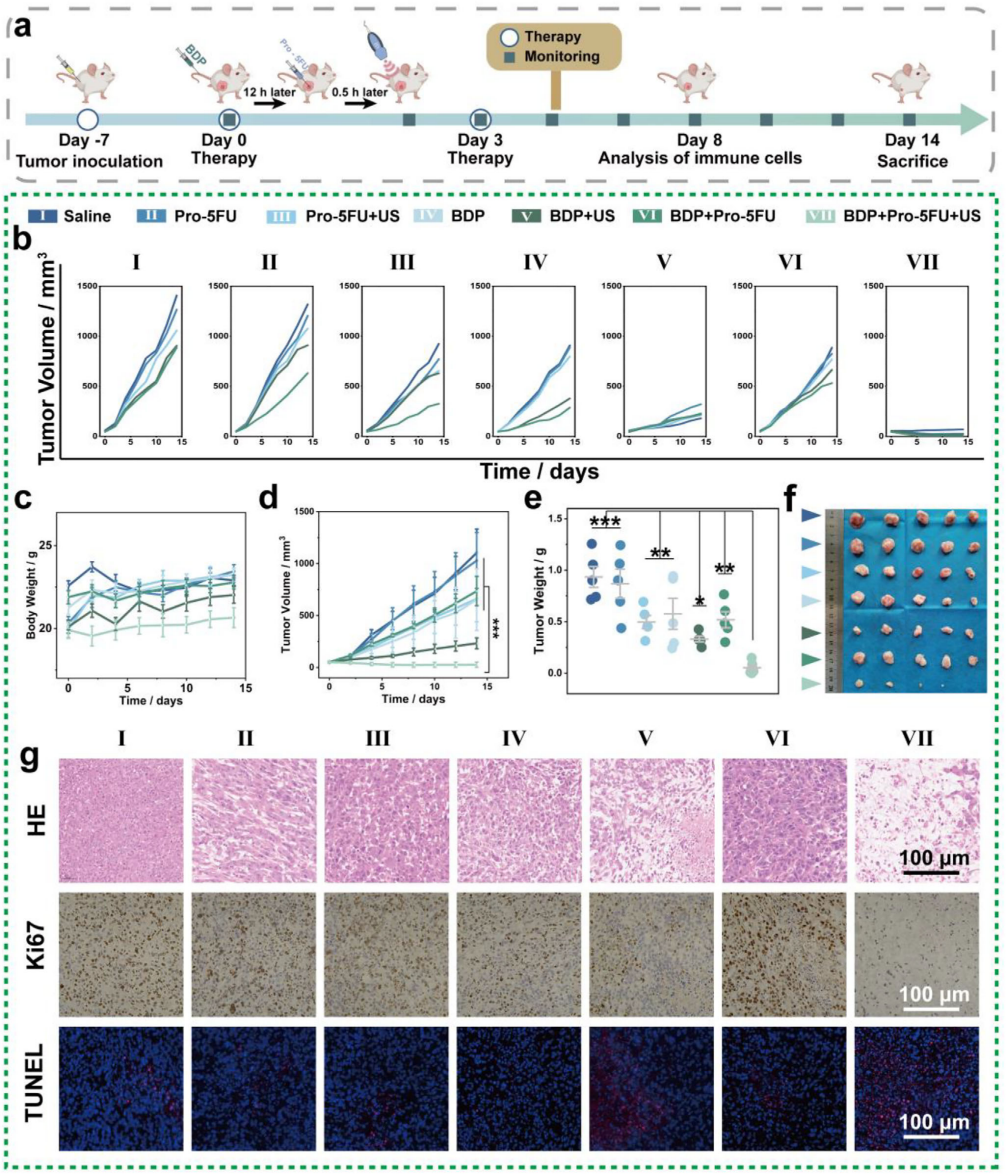
Switchable nanocatalysis for in vivo tumor therapy. (a) Schematic illustration of the animal experimental design. (b) Tumor growth curves of individual mice in different groups. (c) Body weight changes of tumor‐bearing mice during treatments. Data were presented as mean ± SD (n = 5). (d) Relative tumor volume from 0 to 14 days during treatments. Data were presented as mean ± SD (n = 5). (e) Tumor weight of mice after treatments. Data were presented as mean ± SD (n = 5). (f) Photographs of ex vivo tumors obtained on the 14th day. (g) H&E, Ki67, and TUNEL staining of tumor tissues collected at the end of the treatments, scale bars: 100 µm. Significance was calculated using one‐way ANOVA and Tukey's multiple comparisons test. ^*^ *p* < 0.05, ^**^ *p* < 0.01, ^***^ *p* < 0.001, n.s. represents no significant difference.

Additionally, digital photographs of the tumor resection sites in mice (Figure 5f) confirmed that the simultaneous injection of BDP and Pro‐5FU under ultrasound guidance exhibited the strongest therapeutic effect. On day 14, hematoxylin‐eosin (H&E), Ki67, and TdT‐mediated dUTP nick‐end labeling (TUNEL) staining were performed on the excised tumor tissues (Figure 5g; Figure S43 and S44). Furthermore, no significant damage was observed in histological sections of major organs after 14 days of treatment (Figure S45). Collectively, these results demonstrate that BDP+Pro‐5FU+US exerts a potent therapeutic effect against tumors.

To further elucidate the systemic efficacy of piezoelectric catalysis‐controlled bioorthogonal therapy, its underlying immunological mechanisms were investigated. The maturation of DCs was quantified by flow cytometry by assessing the proportion of CD11c^+^, CD80^+^, and CD86^+^ cells in tumor‐draining lymph nodes (TDLNs) in each group. The results in Figure 6a,b, and S46 showed that the percentage of mature DCs reached 37.4% in the BDP+Pro‐5FU+US group, which was significantly higher than that observed in the other groups. Mature dendritic cells (DCs) play a central role in antigen presentation and subsequent activation of adaptive immune responses. As shown in Figure 6c–e and Figure S47, flow cytometry analysis revealed that after BDP+Pro‐5FU+US treatment, Flow cytometry analysis revealed that, following BDP+Pro‐5FU+US treatment, the proportions of CD4^+^ and CD8^+^ T cells increased to 27.5% and 13.2%, respectively. These values were significantly higher than those observed in the BDP+US group (11.4% CD4^+^ and 5.86% CD8^+^ T cells) and the saline control group (3.09% CD4^+^ and 2.06% CD8^+^ T cells). As revealed by immunofluorescence images, the γ‐H_2_AX activation, HMGB1 release, and CRT exposure were found in BDP+US and BDP+Pro‐5FU+US groups. The strongest γ‐H_2_AX signal (Figure 6f,g), the lowest nuclear HMGB1 fluorescence (Figure 6h,i), and the most pronounced surface exposure of CRT (Figure 6j,k) were observed in the BDP+Pro‐5FU+US‐treated group, indicating that piezoelectric catalysis‐controlled bioorthogonal therapy effectively promotes the induction of ICD. The infiltration of antitumor immune cells was further investigated following treatment completion. The immunofluorescence staining results in Figure 6l–o indicate that BDP+Pro‐5FU+US significantly enhanced the infiltration of CD8^+^ (green signal) and CD4^+^ (red signal) T cells into tumor tissues. Collectively, these results establish that the ultrasound‐triggered piezocatalytic bioorthogonal system elicits a robust antitumor immune response and significantly enhances immunotherapeutic efficacy.

**FIGURE 6:**
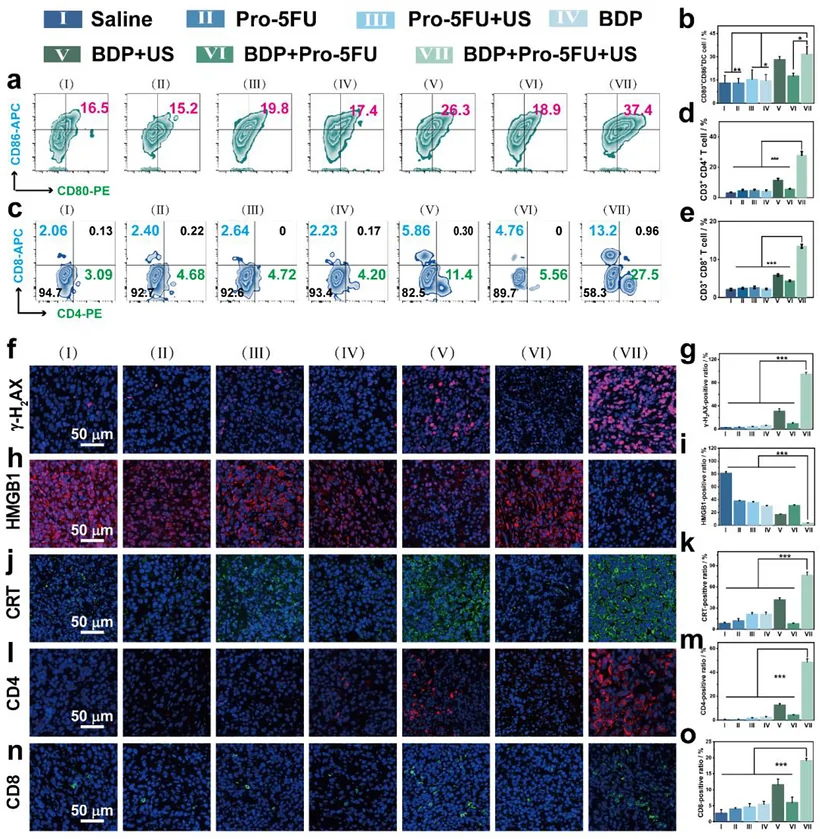
Analysis of immune responses in vivo. (a, b) Representative flow cytometric images and quantitative results of DCs (CD11c^+^, CD80^+^, CD86^+^) in tumor‐draining lymph nodes. Data were presented as mean ± SD (n = 5). (c–e) Representative flow cytometry images and quantitative results of CD4^+^ cells and CD8^+^ T cells in tumors. Data were presented as mean ± SD (n = 5). Representative immunofluorescent staining images and quantitative results of γ‐H_2_AX (f, g), HMGB1 (h, i), CRT (j, k), CD4^+^ (l, m), and CD8^+^ (n, o) in tumors. Data were presented as mean ± SD (n = 5). Significance was calculated using one‐way ANOVA and Tukey's multiple comparisons test. ^*^ *p* < 0.05, ^**^ *p* < 0.01, ^***^ *p* < 0.001, n.s. represents no significant difference.

To further extend the applicability of our system and increase the possibility of future clinical translation, we evaluated the therapeutic efficacy of the piezoelectric bioorthogonal catalytic system via systemic administration by intravenous injection, while keeping the drug concentration unchanged relative to previous experiments. As shown in Figure S48, tumor growth, ex vivo tumor weights, representative tumor images, and mouse body weight were monitored throughout treatment. The ultrasound‐recharging‐regulated closed‐loop bioorthogonal system combined with systemic prodrug administration produced significant tumor growth inhibition compared with control groups, without noticeable body weight loss. These results demonstrate that the piezoelectric bioorthogonal catalytic platform enables effective and well‐tolerated systemic prodrug activation in vivo, supporting its potential for clinically relevant cancer therapy.

## 3 Conclusion

In summary, we have developed an ultrasound‐regulated bioorthogonal catalytic system based on a BaTiO_3_‐Pd heterostructure, which features a smart closed‐loop cycle of on‐demand activation, enhanced catalysis, and self‐termination. This system leverages the pyroelectric effect of BaTiO_3_ for the directed deposition of Pd clusters, constructing an initially silent catalytic interface. Upon ultrasound stimulation, the piezoelectric effect takes over: the generated built‐in electric field reduces Pd^2+^ to active Pd^0^, switching the bioorthogonal reaction ON, while the concomitant external field drastically accelerates the catalytic kinetics. Crucially, after the ultrasound is turned off, the piezocatalytically produced ROS automatically reoxidizes Pd^0^ back to Pd^2+^, switching the system OFF and completing a single‐stimulus‐initiated closed loop. This design ensures spatiotemporally precise and self‐limiting prodrug activation at the tumor site, synergizing with localized ROS generation to potentiate immunogenic cell death and antitumor immunity. This work establishes a safe, controllable, and efficient new paradigm for bioorthogonal catalytic therapy, addressing the critical barrier of dose precision for its clinical translation. Furthermore, it provides a foundational design principle for developing intelligent, mechano‐responsive therapeutic platforms in precision oncology.

## 4 Experimental Section

### 4.1 Materials

Ba(NO_3_)_2_ (barium nitrate), Ti(Bu)_4_ (tetrabutyl titanate), NaOH, 1‐butanol solution, oleic acid(OA), Na_2_PdCl_4_, cyclohexane, dihydrocaffeic acid, tetrahydrofuran (THF), and ethanol were purchased from Shanghai Aladdin Biochemical Technology Co., Ltd. (China). COP‐1 was purchased from Xi`an Ruixi Biological Technology Co., Ltd. All chemicals were used as received without further purification. Deionized water was used for all experiments.

### 4.2 Synthesis of Hydrophobic Ultrasmall Barium Titanate Nanocrystals (BA NPs)

Barium titanate nanoparticles were synthesized via a hydrothermal reaction. An aqueous solution (10 mL) containing 2 mm Ba(NO_3_)_2_ and 1 g NaOH was mixed. The mixture was combined with 5 mL oleic acid (OA) and 20 mL of a 1‐butanol solution containing 2 mm Ti(Bu)_4_ in a 100 mL autoclave under continuous stirring. The reactor was sealed and maintained at 180°C for 48 h. After naturally cooling to room temperature, the products were collected by centrifugation using ethanol and subsequently redispersed in cyclohexane, affording a stable milky colloidal dispersion [34, 50].

### 4.3 Synthesis of Hydrophilically Modified Ultrasmall Barium Titanate Nanocrystals (BD NPs)

Dihydrocaffeic acid (50 mg) was dissolved in 6 mL of THF in a 25 mL three‐neck flask. Subsequently, 20 mg of hydrophobic BA nanoparticles dispersed in 1 mL of THF were added dropwise under stirring. The mixture was heated and maintained for 3 h, followed by cooling to room temperature. Then, 500 µL of NaOH solution (0.5 M) was introduced to induce precipitation of the ultrasmall nanoparticles. The precipitate was collected by centrifugation (3000 rpm), redispersed in 2 mL of water, and stored for subsequent use [51].

### 4.4 Synthesis of Piezoelectric Bioorthogonal System (BDP NPs)

A homogeneous mixture was prepared from 10 mL of the prepared BD NPs solution (1 mg mL^−1^) and 10 mL of a 1 mM Na_2_PdCl_4_ solution. This mixture was transferred to a 50 mL centrifuge tube and subjected to 10 thermal cycles (each cycle: heating to 60°C for 10 min, followed by cooling to 0°C for 10 min). Then, the dispersion was purified by ultrafiltration against deionized water using a 3500 kDa molecular weight cutoff (MWCO) dialysis bag for 3 days, and the products were recovered by freeze‐drying.

### 4.5 Synthesis of Pro‐5FU

5‐Fluorouracil (200 mg, 1.54 mmol) and 1,8‐diazabicyclo[5.4.0]undec‐7‐ene (DBU, 269 µL, 1.80 mmol) were dissolved in anhydrous DMF (2 mL) under a nitrogen atmosphere and cooled to 4°C. Propargyl bromide (1.54 mmol) was separately dissolved in anhydrous DMF (0.5 mL) and added dropwise to the reaction mixture. The resulting solution was stirred at room temperature overnight. After completion of the reaction, the solvent was removed under reduced pressure, and the crude product was purified by flash column chromatography (3% MeOH in DCM) [25].

^1^H NMR (400 MHz, DMSO) δ (ppm): 11.91 (d, J = 3.9 Hz, 1H), 8.13 (d, J = 6.6 Hz, 1H), 4.46 (d, J = 2.5 Hz, 2H), 3.44 (s, 1H).

### 4.6 Characterization of BA NPs, BD NPs, and BDP NPs

The microstructure of BA NPs, BD NPs, and BDP NPs was observed by transmission electron microscopy (TEM, Talos F200S), and high‐resolution TEM (HRTEM, Talos F200S), and the types of elements were analyzed using an energy‐dispersive spectrometer (EDS). And the diameter of the BA NPs was measured manually according to the TEM images. The information on the composition and crystal phase structure of BD NPs was obtained by X‐ray diffraction (XRD, Rigaku Ultima IV, Japan) analysis and Raman spectra (Renishaw in Via, UK). Piezoelectric microscopy (PFM) tests were conducted using atomic force microscopy (AFM, OXFORD). The oxidation potential was determined by differential pulse voltammetry (DPV) to assess the reduction capability of allomelanin nanoparticles. Measurements were performed using a CHI660D electrochemical workstation with a platinum wire as the counter electrode and an Ag/AgCl electrode as the reference electrode. The Zeta potential was measured using zeta particle size potentiostat (Malvern, UK). The valence band position was analyzed by X‐ray photoelectron spectroscopy (XPS, Thermo Scientific K‐Alpha, USA). A UV−vis spectrometer (UV, Shimadzu UV‐3600i Plus, Japan) was used to measure the bandgap of BDP NPs. The conduction band position was measured using an electrochemical workstation (Shanghai Hualong, China).

### 4.7 Density Functional Theory Calculations

All density functional theory (DFT) calculations were carried out using the Vienna Ab initio Simulation Package (VASP) [63]. The electron exchange–correlation interactions were described within the generalized gradient approximation (GGA) using the Perdew–Burke–Ernzerhof (PBE) functional, [64] while the ion–electron interactions were treated using the projector augmented‐wave (PAW) method. A plane‐wave kinetic energy cutoff of 500 eV was applied. Structural optimizations were considered converged when the Hellmann–Feynman forces on each atom were below 0.02 eV Å^−^^1^ and the total energy change between successive ionic steps was less than 10^−^^5^ eV. A vacuum layer of 20 Å was introduced perpendicular to the heterostructure to avoid spurious interactions between periodic images. Polarization calculations were performed using the Berry phase approach as implemented in VASP.

### 4.8 Detection of ·OH, ·O_2_^−^, and Total ROS

·OH, ^·^O_2_^−^, and ROS were detected by TA, NBT, and DCFH methods, respectively. 100 µL of the sample suspension (final concentration: 100 µg mL^−1^) was added to the water, and 400 µL of TA (1 mM), 200 µL of NBT (2 mg mL^−1^), or 10 µL of DCFH (1 mM) were added to make a final volume of 1 mL. The above mixtures were then exposed to ultrasound (1.0 W cm^−2^, 50%, 1 MHz), and the reaction solutions were analysed at different time points. The change in absorbance of NBT at 560 nm or fluorescence of TA/DCF at about 425/525 nm (excitation light: 315/488 nm) was detected.

### 4.9 Monitoring the Uncaging of the Prodrug Pro‐5FU

In vitro validation of bioorthogonal and piezoelectric catalytic properties: To verify the BDP‐mediated elimination reaction by HPLC, BDP dispersion (1 mg mL^−^^1^) was added to 10 mL of PBS containing 500 µm Pro‐5FU and sonicated for different time periods (1.0 MHz, 1.0 W cm^−^^2^, 50% duty cycle). After sonication, the reaction mixture was directly separated via HPLC on a SHIMSEN Ankylo C18‐AQ column (4.6 × 100 mm, 5 µm) using a water/methanol gradient (50% additive‐specify) at 1.0 mL min^−^^1^ for 15 min. Detection employed a PDA detector and a 265 nm detector.

### 4.10 Cell Lines

4T1 Mouse Breast Cancer Cell Line (OriCell, RRID: CVCL 0125) was purchased from Cyagen Biosciences (Guangzhou) Inc. and cultured in high‐glucose DMEM supplemented with 10% fetal bovine serum and 1% penicillin–streptomycin (Thermo Fisher Scientific, USA) at 37°C in a humidified incubator containing 5% CO_2_.

L929 Mouse Fibroblast Cell Line (OriCell, RRID: CVCL 0462) was also obtained from Cyagen Biosciences (Guangzhou) Inc. and maintained in RPMI‐1640 medium supplemented with 10% fetal bovine serum and 1% penicillin–streptomycin under the same incubation conditions (37°C, 5% CO_2_).

### 4.11 Evaluation of Cytotoxicity and Cellular Uptake

4T1 cells were seeded in 96‐well plates at a density of 1.0 × 10^4^ cells per well. Cells were then incubated with different concentrations of BDP NPs or Pro‐5FU for 24 h. Subsequently, 10% CCK‐8 solution was added to each well and incubated at 37°C for 2 h. The absorbance at 450 nm was measured and normalized to the control group.

4T1 cells were planted in 12‐well plates and incubated with Cy7‐BDP NPs (100 µg mL^−1^) for 1, 2, and 4 h. Then, the cells were washed three times with PBS and incubated with Hoechst 33342 (Beyotime, China) for 10 min. Subsequently, the cells were imaged by confocal laser scanning microscopy (Leica TCS SP8).

4T1 cells were seeded in confocal dishes at 1.5 × 10^5^ cells per well. After 24 h, cells were treated with PBS (control), BD, or BDP (100 µg mL^−1^, 4 h) and washed with PBS. Cells were incubated with COP‐1 (30 min) and washed with PBS. Regional ultrasound irradiation was applied using an ultrasound apparatus (Chattanooga 2776, USA) at a power density of 1.0 W cm^−^^2^, a duty cycle of 50%, and a frequency of 1 MHz for 5 min. The treated samples were subsequently analyzed by confocal laser scanning microscopy (CLSM) and flow cytometry (λex = 488 nm, λem = 525 nm).

4T1 cells were seeded in confocal dishes at 1.5 × 10^5^ cells per well. After 24 h, cells were treated with PBS, BD, or BDP (100 µg mL^−1^, 4 h) and washed with PBS. Cells were sonicated (1 W cm^−2^, 50%, 1 MHz, 5 min), incubated with DCFH‐DA (10 µm, 30 min), washed with PBS, and analyzed by CLSM and flow cytometry (λex = 488 nm, λem = 525 nm) to quantify BDP‐induced ROS under ultrasound.

### 4.12 Piezoelectric‐Enabled Orthogonal Prodrug Conversion on Cells

4T1 cells were seeded at 1 × 10^6^ per well in 6‐well plates and cultured overnight. The medium was replaced with fresh medium containing 500 µm prodrug and 200 µg mL^−^^1^ BDP, followed by incubation for 4 h. Cells were divided into ultrasound and non‐ultrasound groups. The ultrasound group received ultrasound stimulation (1.0 MHz, 1.0 W cm^−^^2^, 50% duty cycle) for indicated durations, while the non‐ultrasound group was treated identically without applying ultrasound. Immediately after stimulation, the medium was removed, and cells were washed twice with ice‐cold PBS. Then, 200 µL of ice‐cold 80% methanol was added to each well. Cells were scraped, transferred to centrifuge tubes, disrupted by ice‐bath ultrasonication or freeze‐thaw cycles, and centrifuged. The supernatants were analyzed by LC‐MS to quantify 5‐FU release and calculate the prodrug activation fraction.

### 4.13 In Vitro Inhibition of Cancer Cells and ICD Evaluation

4T1 cells were seeded in 96‐well plates at a density of 8 × 10^3^ cells per well and allowed to adhere overnight. The cells were then incubated with different concentrations of BDP for 24 h and subsequently washed with PBS. Ultrasound treatment (1 W cm^−^^2^, 50% duty cycle, 1 MHz) was applied for 5 min, followed by another PBS wash. Subsequently, 10 µL of CCK‐8 solution and 100 µL of fresh culture medium were added to each well and incubated for an additional 1 h. The absorbance at 450 nm was finally measured using a microplate reader.

To further evaluate cancer cell‐killing efficacy, live/dead staining and flow cytometry analyses were performed. 4T1 cells were seeded in confocal dishes at a density of 1.5 × 10^5^ cells per well. After 24 h of culture, the cells were incubated with PBS, Pro‐5FU, BDP, or BDP + Pro‐5FU for 4 h, followed by washing with PBS. For the PBS + US, Pro‐5FU + US, BDP + US, and BDP + Pro‐5FU + US groups, regional ultrasound irradiation was applied using an ultrasound apparatus at 1.0 W cm^−^^2^, 50% duty cycle, and 1 MHz for 5 min. All groups were subsequently co‐stained with FDA and PI for 30 min and washed with PBS before imaging using an inverted fluorescence microscope (IX‐71, Olympus). For apoptosis analysis, cells treated under identical conditions were stained with Annexin V–FITC and PI and analyzed by flow cytometry (CytoFlex, Beckman Coulter, USA).

Samples from cultured cells were prepared following the same procedure. The cells were stained with aggregates and monomer dyes to assess changes in mitochondrial membrane potential, with caspase‐3 staining to evaluate caspase‐3 activation, and with γ‐H_2_AX staining to detect DNA damage. The stained cells were then imaged using an inverted fluorescence microscope. In addition, cells were stained with HMGB1 and CRT for immunogenic cell death (ICD) evaluation and analyzed by flow cytometry.

### 4.14 Dendritic Cells (DCs) Maturation

Bone‐marrow‐derived dendritic cells (BMDCs) were generated according to a previous study [52]. Briefly, the mouse bone marrow cells were incubated in the RPMI‐1640 medium supplemented with IL‐4 (10 ng mL^−1^) and GM‐CSF (10 ng mL^−1^) for 7 days to obtain immature DCs. The culture medium was changed every 3 days.

The induction of DCs maturation was evaluated by a transwell co‐culture system, where the upper chamber was cultured with 4T1 cells with a cell density of 1.5 × 10^5^ cells, and the lower chamber was seeded with DCs at a cell density of 2 × 10^5^ cells. After 24 h, cells were cultured with PBS, Pro‐5FU, BDP, and BDP+Pro‐5FU for 4 h, and washed with PBS. For PBS+US, Pro‐5FU+US, BDP+US, and BDP+Pro‐5FU+US groups, a regional ultrasound was applied by an ultrasound apparatus at 1.0 W cm^−2^, 50%, 1 MHz for 5 min. Treated 4T1 cells were co‐cultured with DC cells for 12 h. After that, the DCs were collected and stained with anti‐CD11c, anti‐CD80, and anti‐CD86 for further flow cytometry detection.

### 4.15 Hemolysis Analysis

Whole blood from healthy Balb/c mice was centrifuged and subjected to five washes to obtain red blood cells (RBCs). Subsequently, the RBCs suspension was incubated with different concentrations of BDP at 37°C for 2 h. PBS solution and deionized water were set as negative and positive control groups, respectively. After incubation, the supernatants were collected by centrifugation at 1500 rpm for 10 min, and the absorbance at 540 nm was measured using a microplate reader.

### 4.16 Blood Circulation of BDP

BDP dispersion (5 mg kg^−1^, approximately 14.5 × 10^12^ particles per mouse) was administered to mice via tail vein injection. At designated time points (3, 6, 12, 24, and 48 h post‐injection), mice (n = 3 per time point) were euthanized, and the heart, liver, spleen, lung, kidney, and tumor tissues were collected. Approximately 0.1 g of each tissue was weighed, minced, and digested with 3 mL of concentrated nitric acid and 1 mL of 30% hydrogen peroxide at 120°C for 2 h. After digestion, the solution was diluted to 10 mL with ultrapure water. The barium (Ba) content in each sample was determined by inductively coupled plasma atomic emission spectrometry (ICP‐AES). The clearance half‐life of BDP in tissues was calculated from the Ba concentration–time curve.

### 4.17 Mouse Tumor Model Establishment

All animal experiments were performed by the regulations of the Institutional Animal Care and Use Committee of Chongqing University (CQU‐IACUC‐RE‐202501‐005). Six‐week‐old female Balb/c mice were purchased from GemPharmatech Co., Ltd.

For evaluation of the anticancer efficacy, subcutaneous tumor models were established by injecting 5 × 10^6^ 4T1 cells into the right mammary fat pad of 6‐week‐old female BALB/c mice. When tumor volumes reached approximately 80–100 mm^3^, in vivo treatments were initiated.

The mice were randomly divided into seven groups: (I) Saline, (II) Pro‐5FU, (III) Pro‐5FU + US, (IV) BDP, (V) BDP + US, (VI) BDP + Pro‐5FU, and (VII) BDP + Pro‐5FU + US. BDP was administered via intravenous injection at a dose of 5 mg kg^−^^1^ in a volume of 100 µL per mouse (corresponding to 1 mg mL^−^^1^ based on an average body weight of 20 g, corresponding to approximately 1.45 × 10^1^ ^3^ particles) [65]. Pro‐5FU was administered by intratumoral injection at 10 mg kg^−^^1^ in a volume of 50 µL per mouse (4 mg mL^−^^1^).

Ultrasound (US) treatment (1.0 W cm^−^^2^, 50% duty cycle, 1 MHz, 5 min) was applied during the first three days of therapy. Body weight and tumor volume were recorded every two days starting from the first treatment day. Tumor volume was calculated using the formula V = L × W^2^ / 2, where L represents the longest tumor diameter and W the shortest diameter.

At the designated endpoint, a subset of mice was sacrificed for tumor collection. Tumor sections were subjected to H&E, TUNEL, Ki67, γ‐H2AX, HMGB1, CRT, CD4^+^, and CD8^+^ staining to evaluate apoptosis, proliferation, DNA damage, immunogenic cell death, and immune infiltration. Major organs, including the heart, liver, spleen, lung, and kidney, were harvested and analyzed by H&E staining for biosafety assessment. In addition, blood samples were collected for biochemical analysis.

### 4.18 Systemic Administration

To further evaluate the antitumor efficacy of systemic administration, an orthotopic breast cancer model was established by injecting 5 × 10^6^ 4T1 cells into the right mammary fat pad of 6‐week‐old female BALB/c mice. When the tumor volume reached approximately 80–100 mm^3^, in vivo treatment was initiated.

Mice were randomly divided into four groups: (1) saline group, (2) Pro‐5FU + US group, (3) BDP + US group, and (4) BDP + Pro‐5FU + US group. BDP was administered via tail vein injection at a dose of 5 mg kg^−1^ in a volume of 100 µL (prepared as a 1 mg mL^−1^ solution based on an average body weight of 20 g, corresponding to approximately 1.45 × 10^1^ ^3^ particles). 12 h after BDP injection, Pro‐5FU was administered via tail vein injection at a dose of 10 mg kg^−1^ in a volume of 50 µL (4 mg mL^−;1^). 10 min after Pro‐5FU injection, ultrasound treatment (power 1.0 W/cm^2^, duty cycle 50%, frequency 1 MHz, duration 5 min) was applied to the mice in the relevant groups. Body weight and tumor volume were recorded every other day starting from the first day of treatment. Tumor volume was calculated using the formula V = L × W^2^ / 2, where L is the longest diameter, and W is the shortest diameter. At the designated endpoint, tumors were excised, weighed, and photographed for treatment evaluation.

### 4.19 Tumor‐Infiltrating Immune Cells Analysis

On day 7, tumor tissues and lymph nodes were harvested from 4T1 tumor‐bearing BALB/c mice following different treatments for immune evaluation. Tumors were minced into small fragments and digested in 5 mL PBS containing collagenase IV (1 mg mL^−^^1^), DNase I (1 U mL^−^^1^), and hyaluronidase (0.1 mg mL^−^^1^) at 37°C for 2 h. The digested tissues were subsequently passed through 70 µm cell strainers to obtain single‐cell suspensions. Cells were blocked with 5% BSA for 20 min and washed three times with PBS containing 1% BSA.

For analysis of tumor‐infiltrating T cells, the tumor‐derived cell suspensions were stained with FITC anti‐CD3, APC anti‐CD8, and PE anti‐CD4 antibodies according to the manufacturer's instructions and analyzed by flow cytometry.

Lymph nodes were gently dissociated in culture medium and filtered through 70 µm nylon mesh filters to obtain single‐cell suspensions. The cells were similarly blocked with 5% BSA for 20 min and washed three times with PBS containing 1% BSA. For evaluation of DC maturation, lymph node cell suspensions were stained with FITC anti‐CD11c, PE anti‐CD80, and APC anti‐CD86 antibodies and subsequently analyzed by flow cytometry.

### 4.20 Statistical Analysis

All data are presented as mean ± standard deviation (SD). Data analysis was performed using ImageJ, Origin 2024, and Graphpad Prism 10.0 software. Statistical differences between data were analyzed using either one‐way or two‐way analysis of variance (ANOVA), where the Tukey HSD test was used for pairwise comparisons. p < 0.05 is considered statistically significant, ^*^ *p* < 0.05, ^**^ *p* < 0.01, ^***^ *p* < 0.001, ^****^ *p* < 0.0001, *p* ≥ 0.05 was considered as non‐significant difference (ns).

## Author Contributions

**Daqing Xia**: conceptualization, methodology, software, data curation, formal analysis, writing – review and editing, writing – original draft, validation, investigation. **Lei Liu**: software, visualization, methodology, writing – original draft, writing – review and editing, data curation. **Lunli Xiang**: methodology. **Zhenqiang Wang**: resources, supervision, writing – original draft, funding acquisition. **Guangxu Fang**: validation, visualization. **Jixi Zhang**: supervision, resources, funding acquisition, conceptualization, project administration. **Shuang Jin**: data curation, formal analysis. **Yunyun Wu**: methodology. **Hongrui Zhu**: supervision. **Chang Yu**: data curation.

## Funding

This work was supported in part by the National Natural Science Foundation of China (NSFC, grant nos. 22475028, 22175027, 22305267). Project supported by Graduate Research and Innovation Foundation of Chongqing, China (Grant No. CYB25057) and the Natural Science Foundation of Chongqing (CSTB2024NSCQ‐MSX1275).

## Conflicts of Interest

The authors declare no conflicts of interest.

## Supporting information




**Supporting File**: advs76338‐sup‐0001‐SuppMat.docx.

## Data Availability

The data that support the findings of this study are available from the corresponding author upon reasonable request.
