# Supplementary material for: Ultrasound‐Recharged Sub‐Nanometer Palladium Catalysts for on‐Demand and Self‐Terminating Bioorthogonal Prodrug Activation in Cancer Therapy
Source: Adv Sci (Weinh). 2026 Jul 1:e76338. Online ahead of print. doi: 10.1002/advs.76338 (PMC13336366; doi:10.1002/advs.76338)
Supplement: Supplementary file 1 — Supporting File: advs76338‐sup‐0001‐SuppMat.docx. [file ADVS-9999-e76338-s001.docx]

Ultrasound-Recharged Sub-Nanometer Palladium Catalysts for On-Demand and Self-Terminating Bioorthogonal Prodrug Activation in Cancer Therapy

Daqing Xia, Lei Liu, Shuang Jin, Guangxu Fang, Chang Yu, Yunyun Wu, Lunli Xiang, Hongrui Zhu, Zhenqiang Wang,* Jixi Zhang*

D. Xia, L. Liu, S. Jin, G. Fang, C. Yu, Y. Wu, L. Xiang, H. Zhu, J. Zhang

Key Laboratory of Biorheological Science and Technology, Ministry of Education, College of Bioengineering, Chongqing University, No. 174 Shazheng Road, Chongqing 400044, China.

E-mail: [jixizhang@cqu.edu.cn](mailto:jixizhang@cqu.edu.cn) (Jixi Zhang)

Z. Wang

Department of Pharmacy, The Second Affiliated Hospital, Army Medical University, No. 183 Xinqiao Road, Chongqing 400037, China.

E-mail: [zqwang@tmmu.edu.cn](mailto:zqwang@tmmu.edu.cn)

Funding: This work was supported in part by the National Natural Science Foundation of China (NSFC, grant nos. 22475028, 22175027, 22305267). Project supported by Graduate Research and Innovation Foundation of Chongqing, China (Grant No. CYB25057) and the Natural Science Foundation of Chongqing (CSTB2024NSCQ-MSX1275).

KEYWORDS: Piezocatalysis · Bioorthogonal Chemistry · Closed-Loop Catalysis · Immunogenic Cell Death

**Supporting Figures**


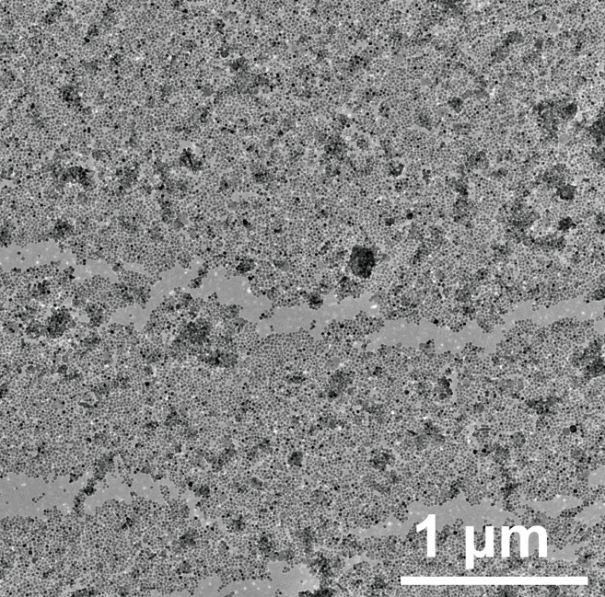


**Figure S1.** TEM image of hydrophobic barium titanate nanoparticles at low magnification.


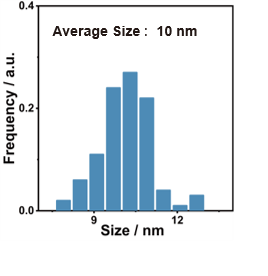


**Figure S2.** The size distribution histogram of BaTiO_3_.


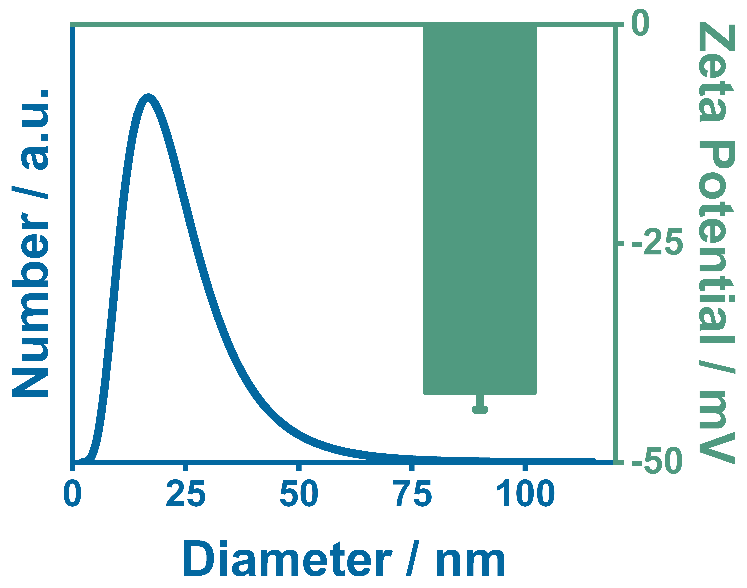


**Figure S3**. Hydrodynamic size distribution and zeta potential of BD. Data were presented as mean ± SD (n = 3).


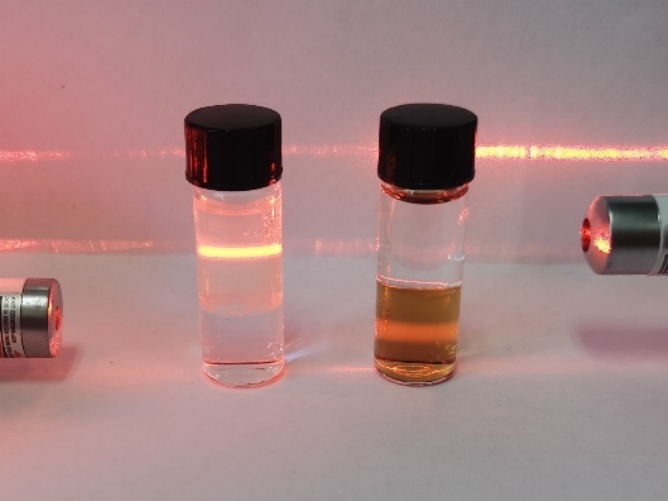


**Figure S4.** Solvent dispersity of BaTiO_3_ before (left) and after (right) ligand exchange.


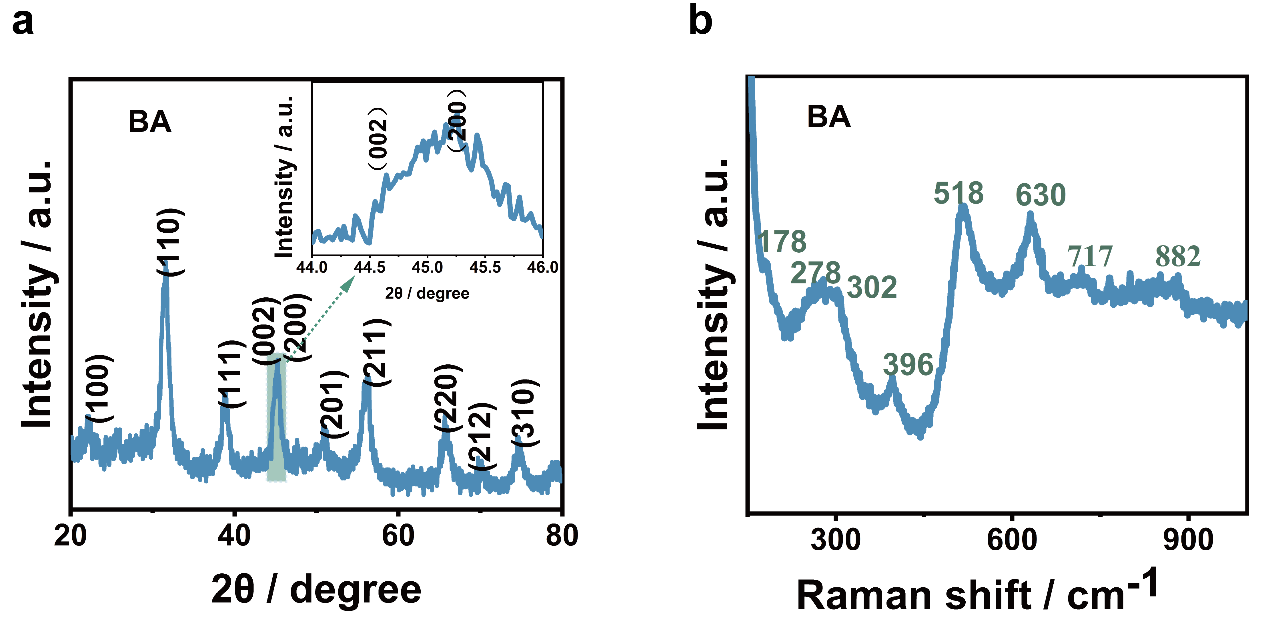


**Figure S5**. XRD pattern (a) and Raman spectrum (b) of BA.


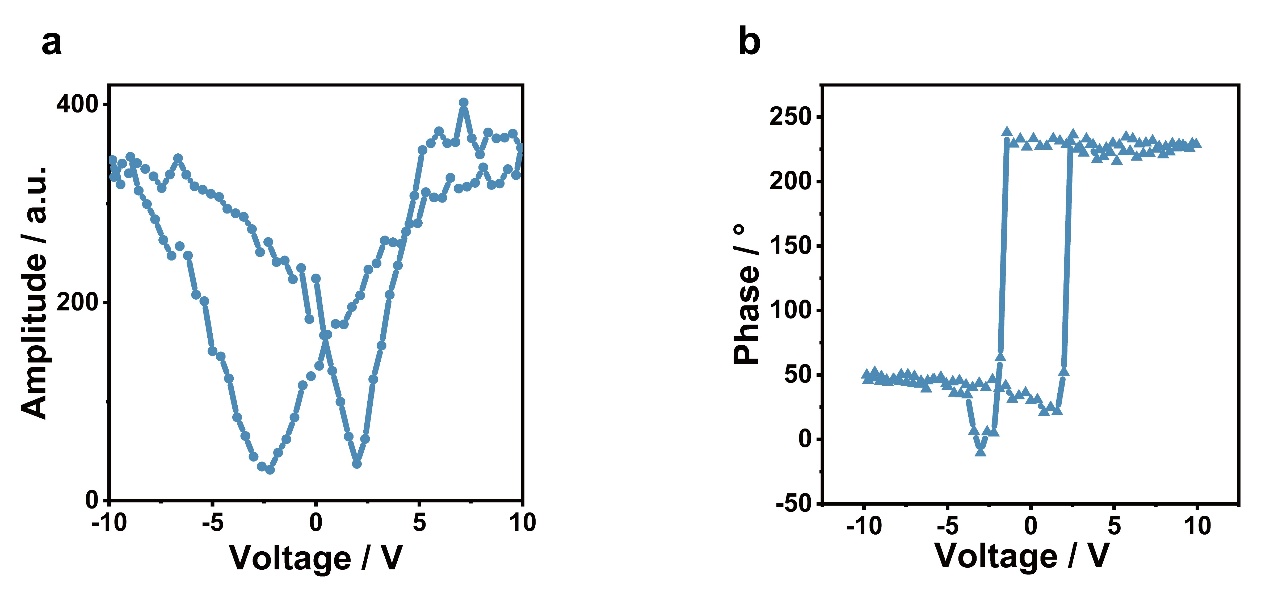


**Figure S6**. Amplitude curve (a) and Phase curve (b) of P-BTO obtained from piezo-response force microscopy.


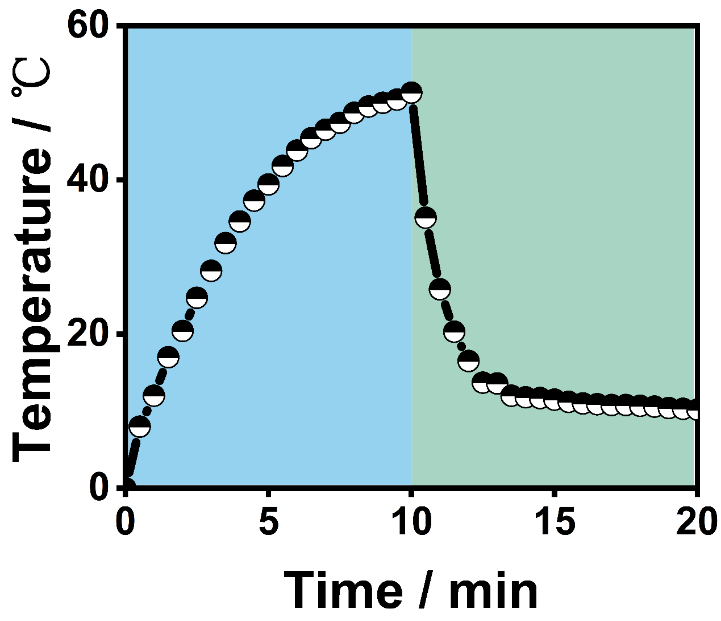


**Figure S7**. The temperature variation of the reaction system during the pyroelectric deposition process.


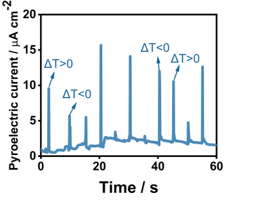


**Figure S8**. Variation of the thermoelectric current of BD under the influence of hot and cold-water oscillation.


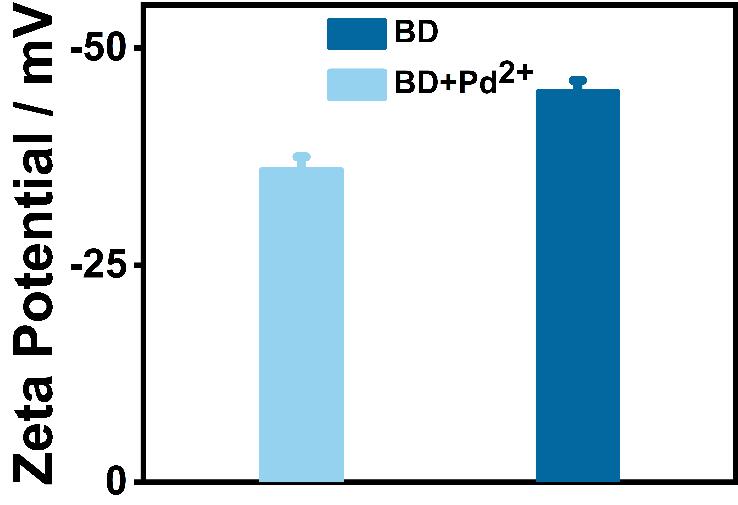


**Figure S9**. Zeta changes before and after the capture of Pd^2+^ by BD. Data were presented as mean ± SD (n = 3).


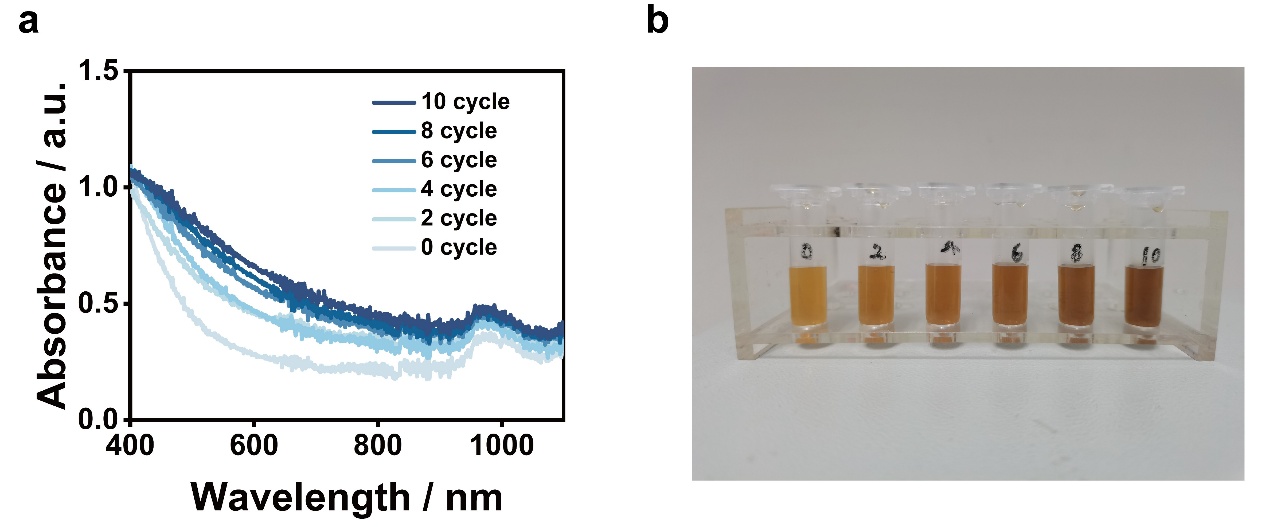


**Figure S10**. The number of thermal-cycle dependent absorbance spectrum changes (a) and color changes (b) of the dispersion solution.


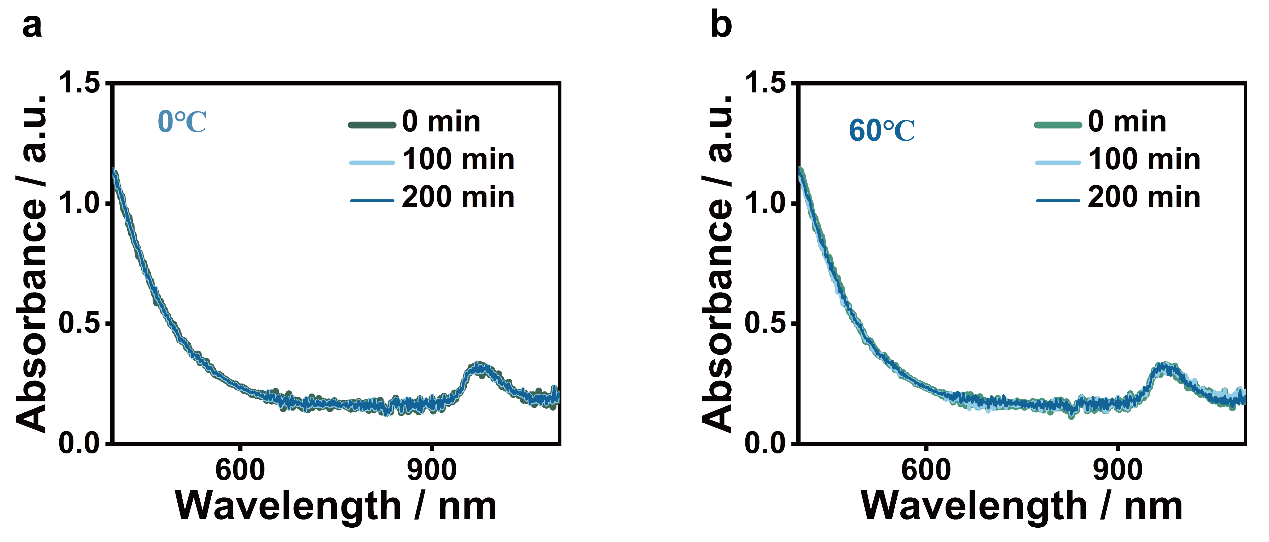


**Figure S11**. Time-dependent absorbance changes of the BD dispersion containing Pd²⁺ at different temperatures: 0°C (a); 60 °C (b).


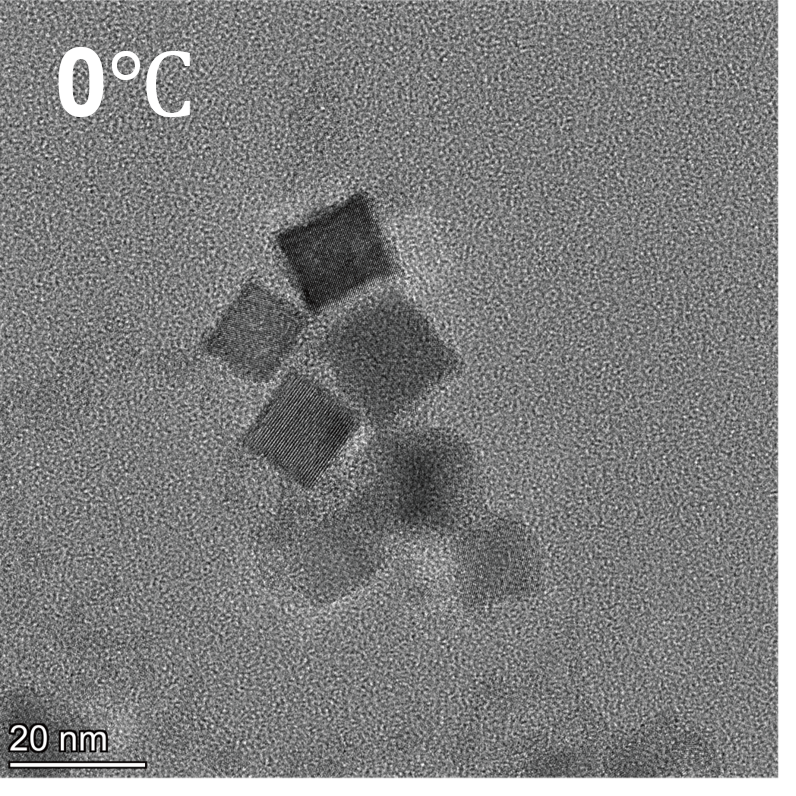


**Figure S12**. TEM image of the mixture of BD and Pd^2+^ after reaction at 0°C for 200 min.


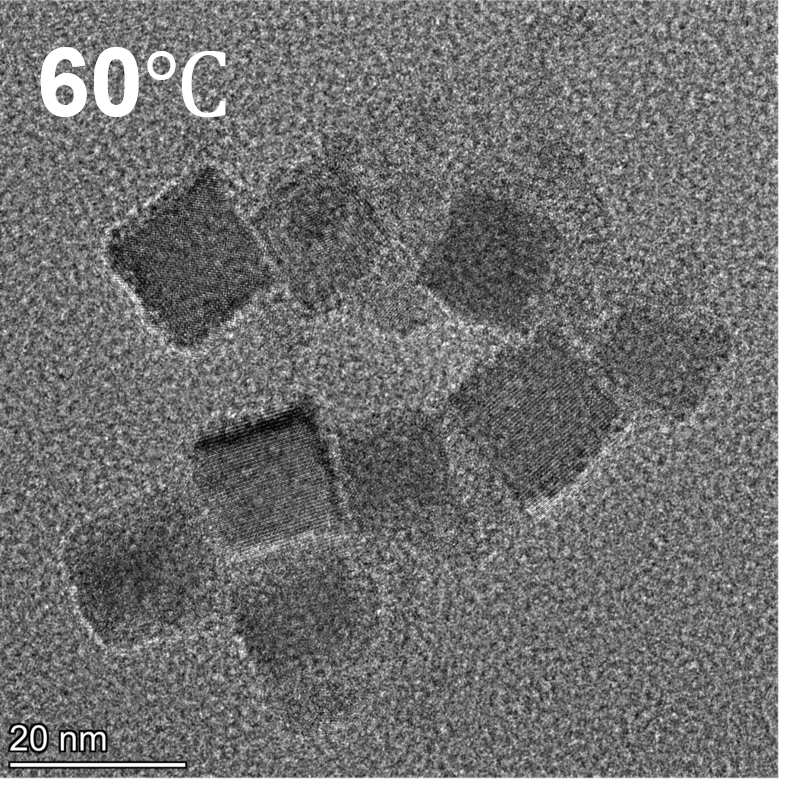


**Figure S13**. TEM image of the mixture of BD and Pd^2+^ after reaction at 60°C for 200 min.


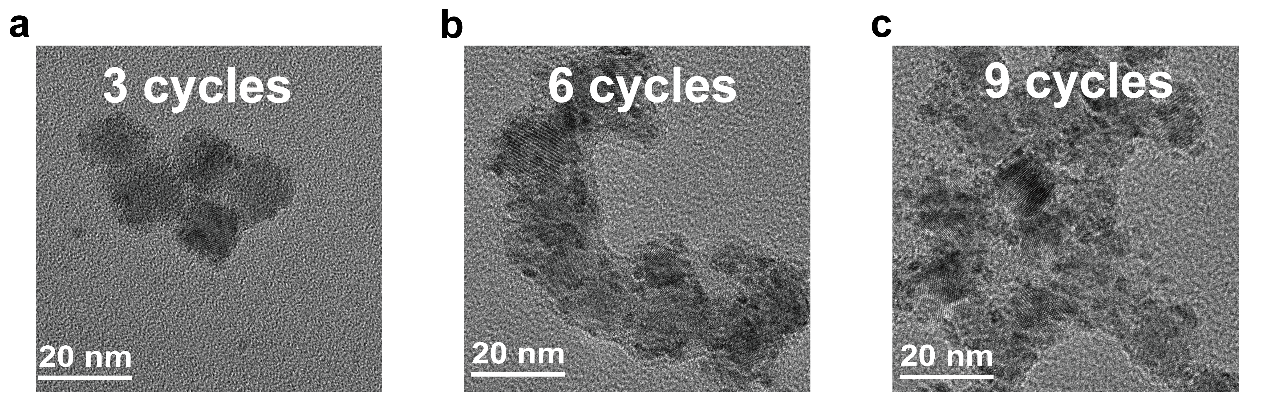


**Figure S14**. TEM images of BDP synthesized through different numbers of thermal cycles (3 cycles (a), 6 cycles (b), and 9 cycles (c)).


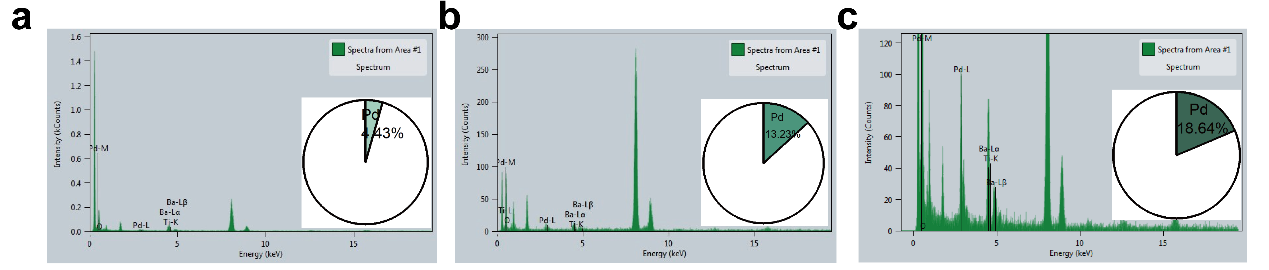


**Figure S15**. TEM-associated energy-dispersive X-ray photoelectron spectroscopy (EDX) data and relative Pd content in BDP. BDP was synthesized through different numbers of thermal cycles (3 cycles (a), 6 cycles (b), and 9 cycles (c)).


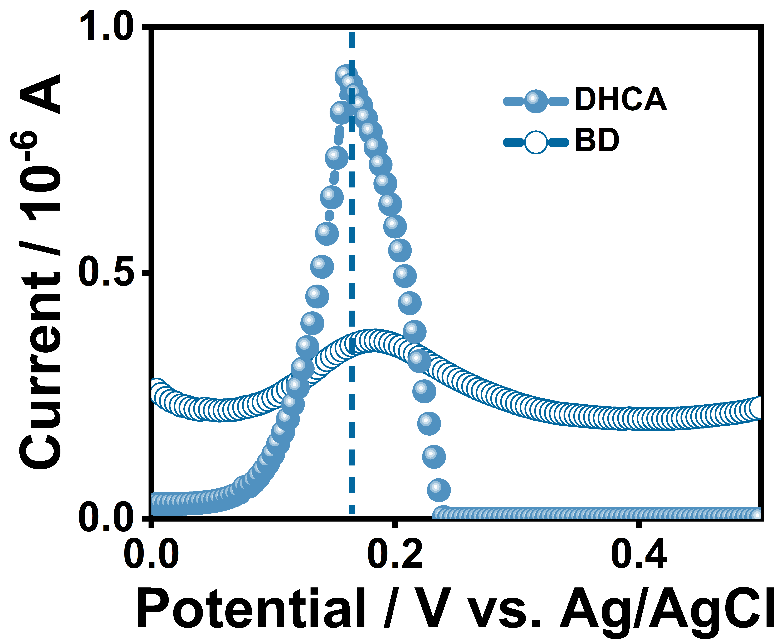


**Figure S16**. DPV curves of DHCA and BD.


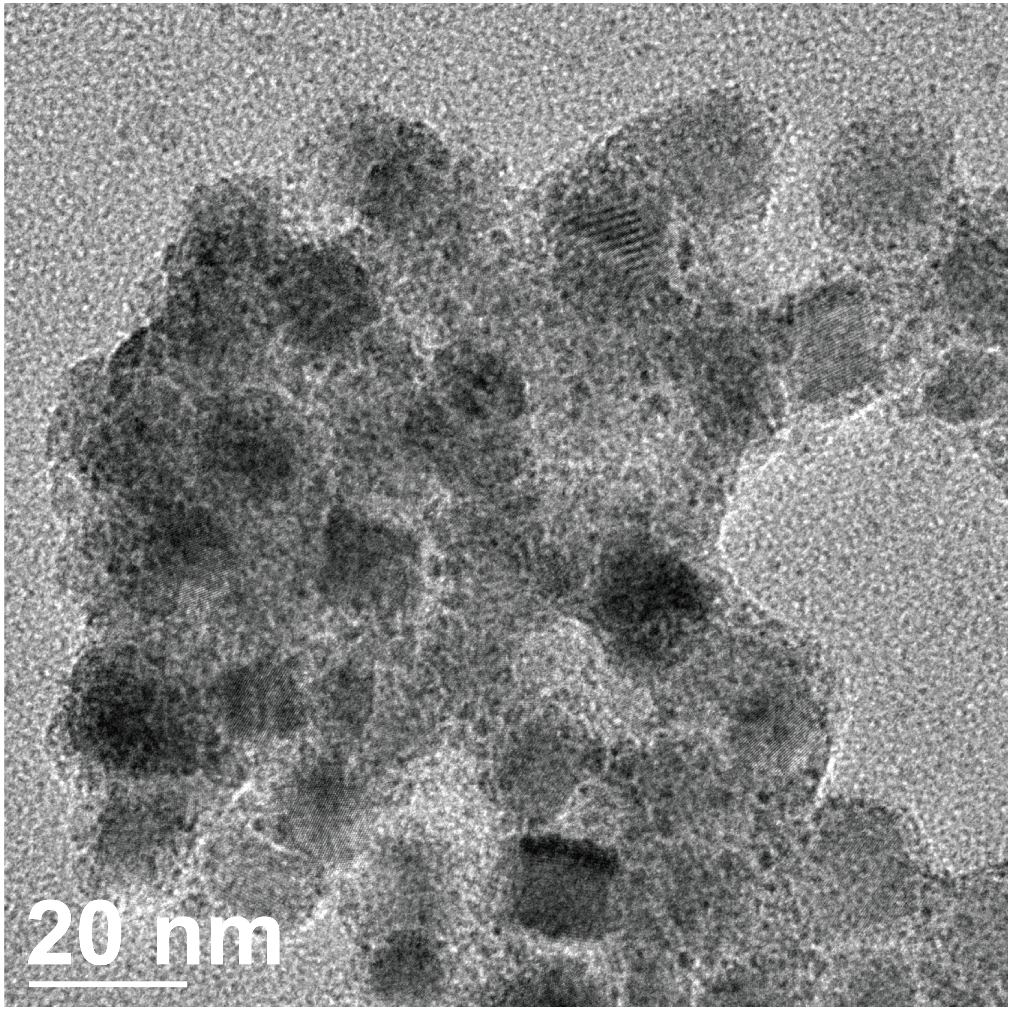


**Figure S17**. TEM image of BDP synthesized by 10 cold-hot cycles under N_2_ protection.


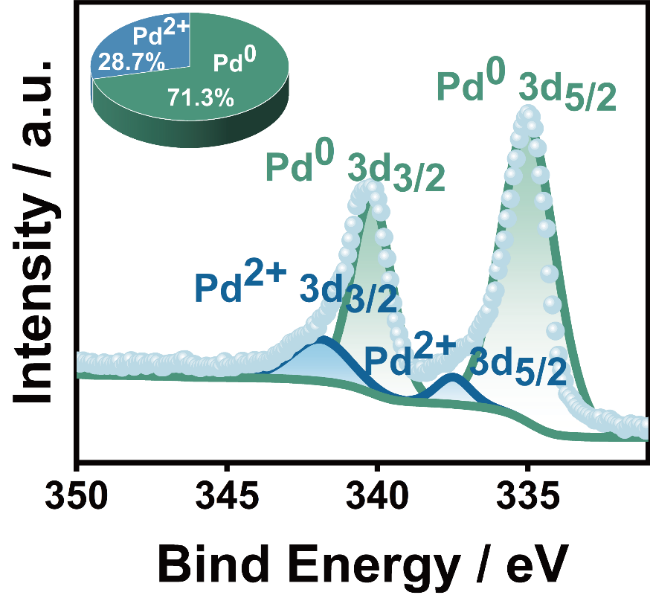


**Figure 18**. XPS spectrum of the Pd 3d region in BDP after 10 thermal cycles under N₂ atmosphere. The inset shows the contents of Pd^2+^ and Pd^0^.


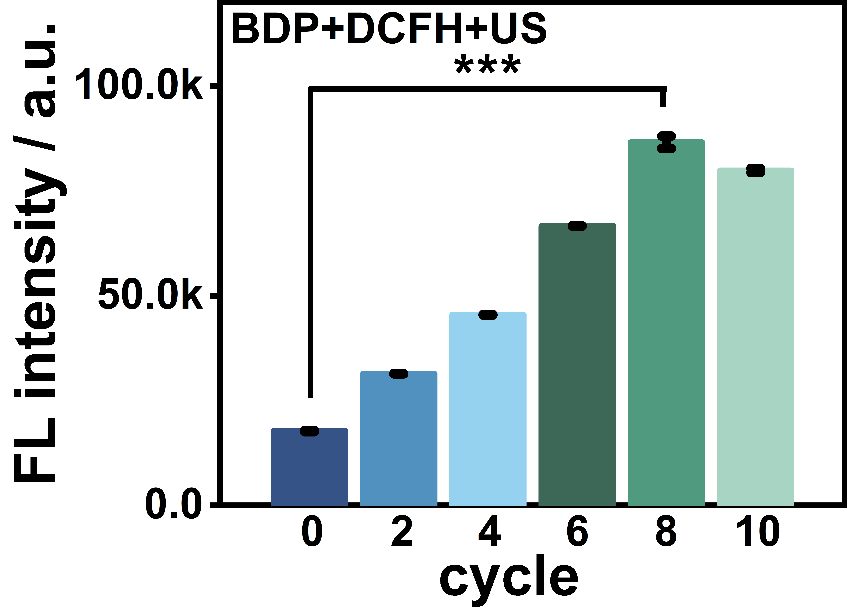


**Figure S19**. Fluorescence intensity of DCFH in the presence of BDP with different numbers of thermal cycles under US irradiation (1.0 MHz, 1.0 W cm⁻², 50% duty cycle, 20 min). Data were presented as mean ± SD (n = 3). Significance was calculated using one-way ANOVA and Tukey’s multiple comparisons test. *P < 0.05, **P < 0.01, ***P < 0.001, n.s. represents no significant difference.


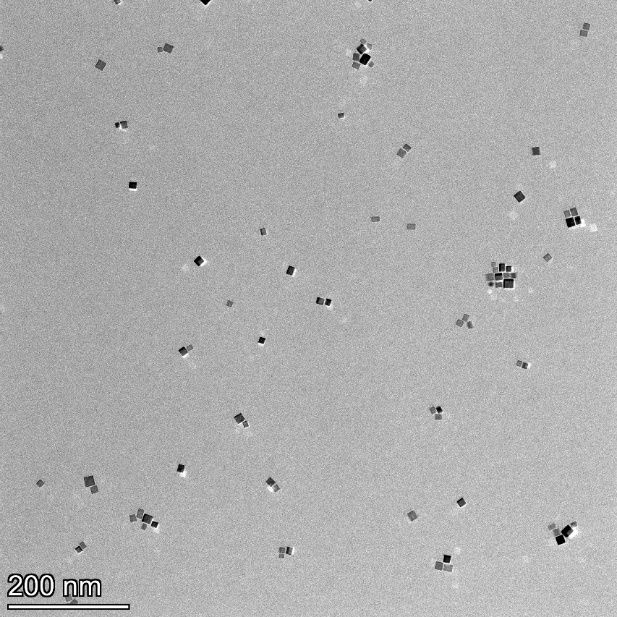


**Figure S20**. TEM image of Pd-PVP.


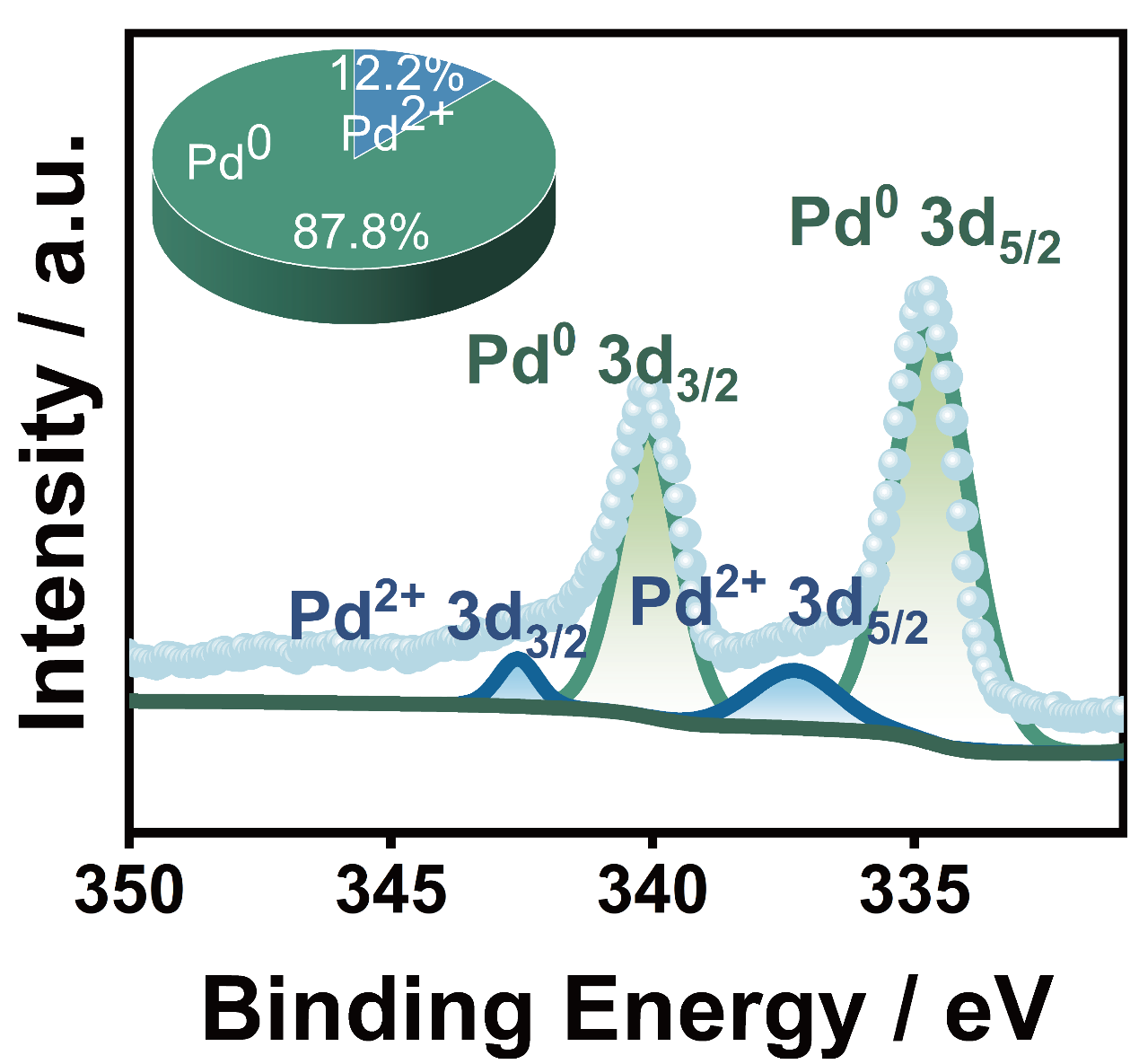


**Figure S21**. XPS spectrum of Pd-PVP. The inset shows the contents of Pd^0^ and Pd^2+^.


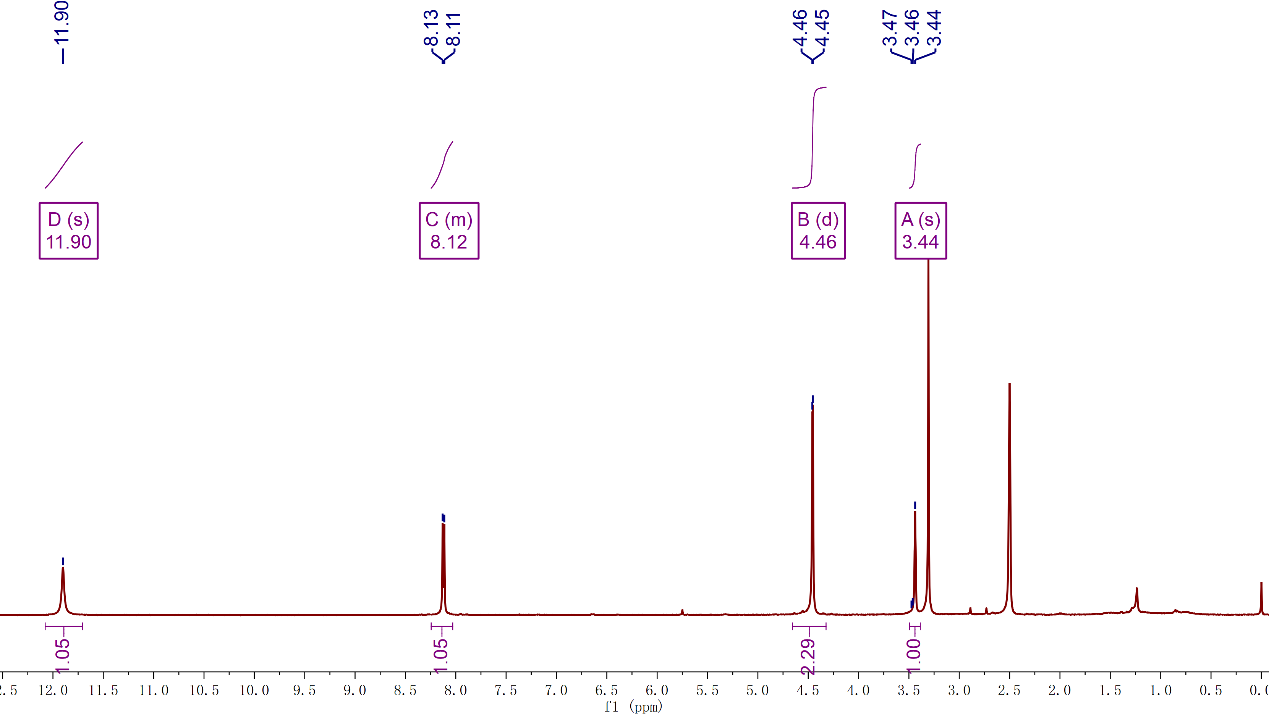


**Figure S22.** ^1^H NMR spectrum of Pro-5FU.


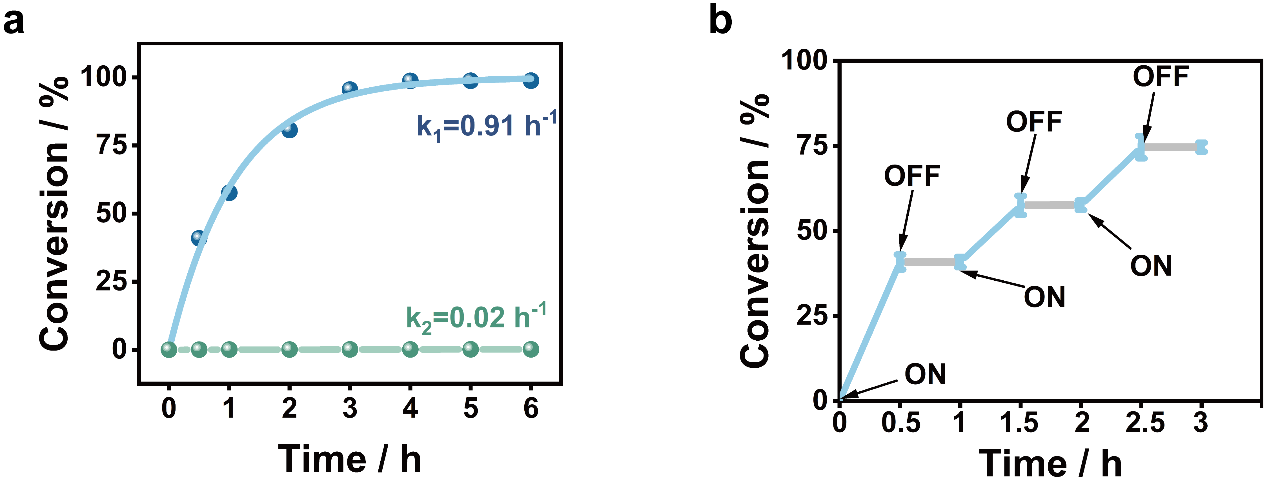


**Figure S23.** Evaluation of the prodrug uncaging efficiency, conversion rate, and cyclic activation/deactivation behavior. (a) Kinetic profiles of prodrug Pro-5FU (500 μM) conversion in the presence of BDP (100 μg mL⁻¹) under ultrasound and non‑ultrasound conditions. (b) Conversion of Pro-5FU under cyclic ultrasound (0.5 h on / 0.5 h off per cycle) in a nitrogen atmosphere, using the same concentrations as in (a) (BDP 100 μg mL⁻¹, Pro-5FU 500 μM). Data were presented as mean ± SD (n = 3).


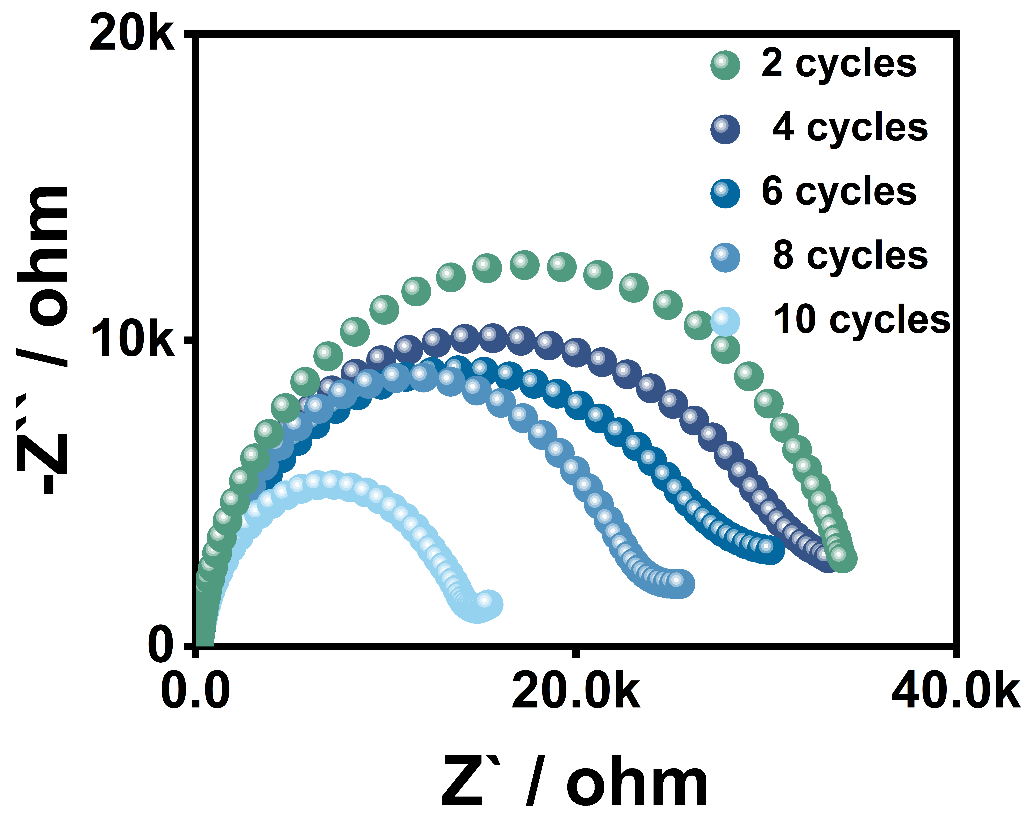


**Figure S24**. Impedance changes of BDP under different numbers of thermal cycles.


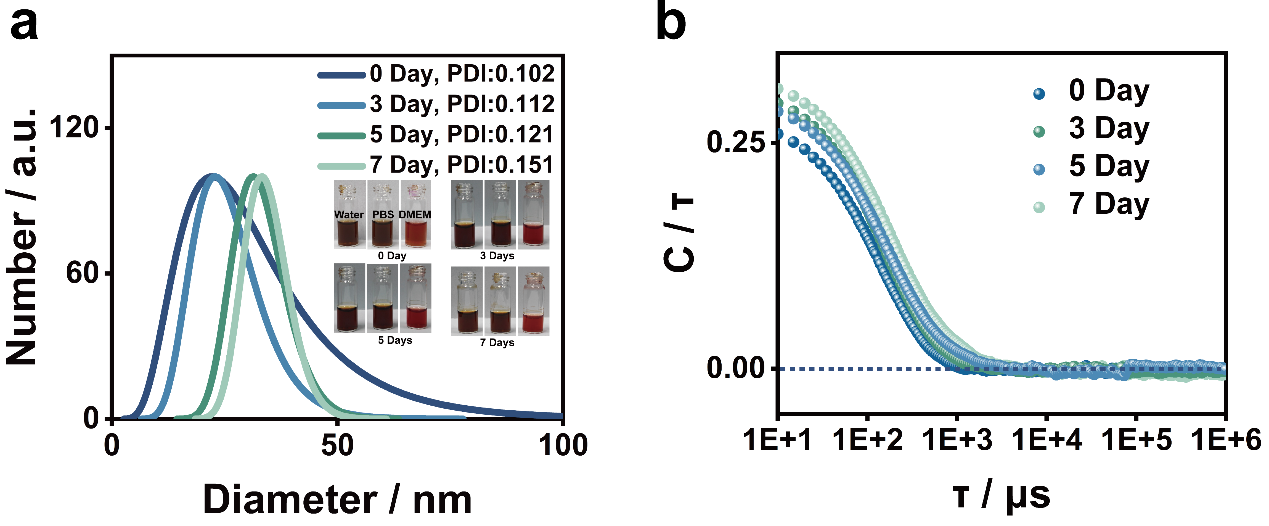


**Figure S25**. (a) Particle-size distributions of BDP after being stored in water for different times. The inset shows BDP dispersed in various solvents. (b) Autocorrelation functions of BDP particles stored in water for different durations.


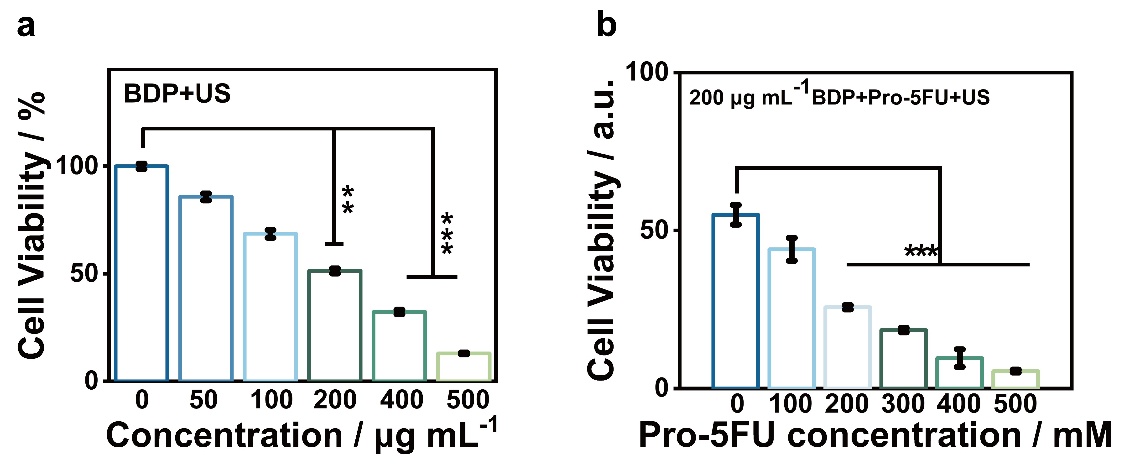


**Figure S26**. Cell viability of 4T1 cells treated with different concentrations of BDP (a) and Pro-5FU (200 μg mL^-1^ BDP+US) (b). Data were presented as mean ± SD (n = 5). Significance was calculated using one-way ANOVA and Tukey’s multiple comparisons test. *P < 0.05, **P < 0.01, ***P < 0.001, n.s. represents no significant difference.


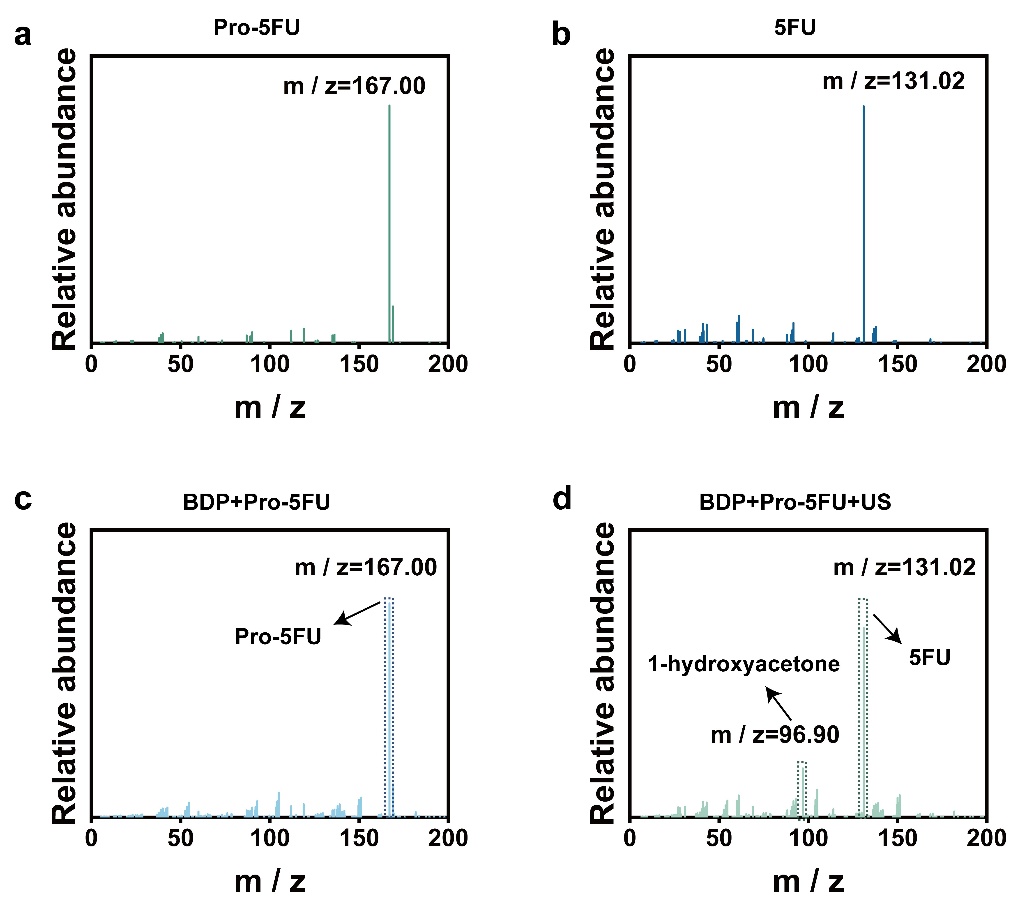


**Figure S27.** Mass spectrometry evidence for piezoelectric bioorthogonal catalytic conversion of a prodrug. a) MS spectrum of Pro‑5FU (m/z [M-H]^-^ = 167.00). b) MS spectrum of 5FU (m/z [M+H]⁺ = 131.02). c, d) MS spectra of cell lysates from BDP + Pro‑5FU (c) without and (d) after 4 h of ultrasound treatment (1.0 MHz, 1.0 W cm⁻², 50% duty cycle).


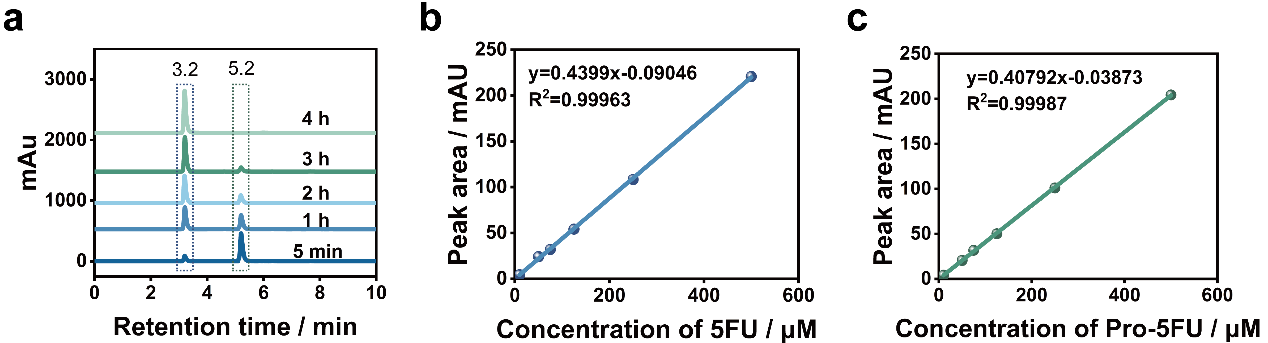


**Figure S28.** LC-MS analysis of intracellular prodrug conversion under different ultrasound treatment durations. (a) LC-MS chromatograms of cell lysates after various ultrasound treatment times, with a particle concentration of 200 μg mL⁻¹ and a final prodrug concentration of 500 μM. (b) and (c) Calibration curves for 5-FU and Pro-5FU, respectively.


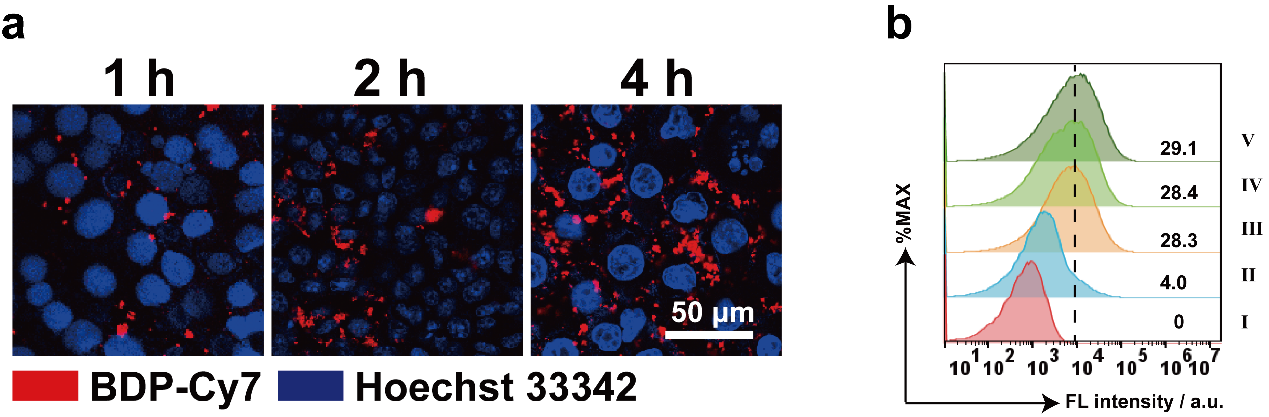


**Figure S29**. (a) CLSM images of 4T1 cells co-cultured with Cy7-labeled BDP for 1, 2, and 4 h. (b) Flow cytometry was used to measure the fluorescence intensity of particles internalized by 4T1 cells within 4 hours in the presence or absence of various inhibitors under the following conditions: (Ⅰ) control, (Ⅱ) BDP-Cy7 + chlorpromazine (20 μM), (Ⅲ) BDP-Cy7 + nystatin (20 μM), (Ⅳ) BDP-Cy7 + EIPA (50 μM), and (Ⅳ) BDP-Cy7 alone.


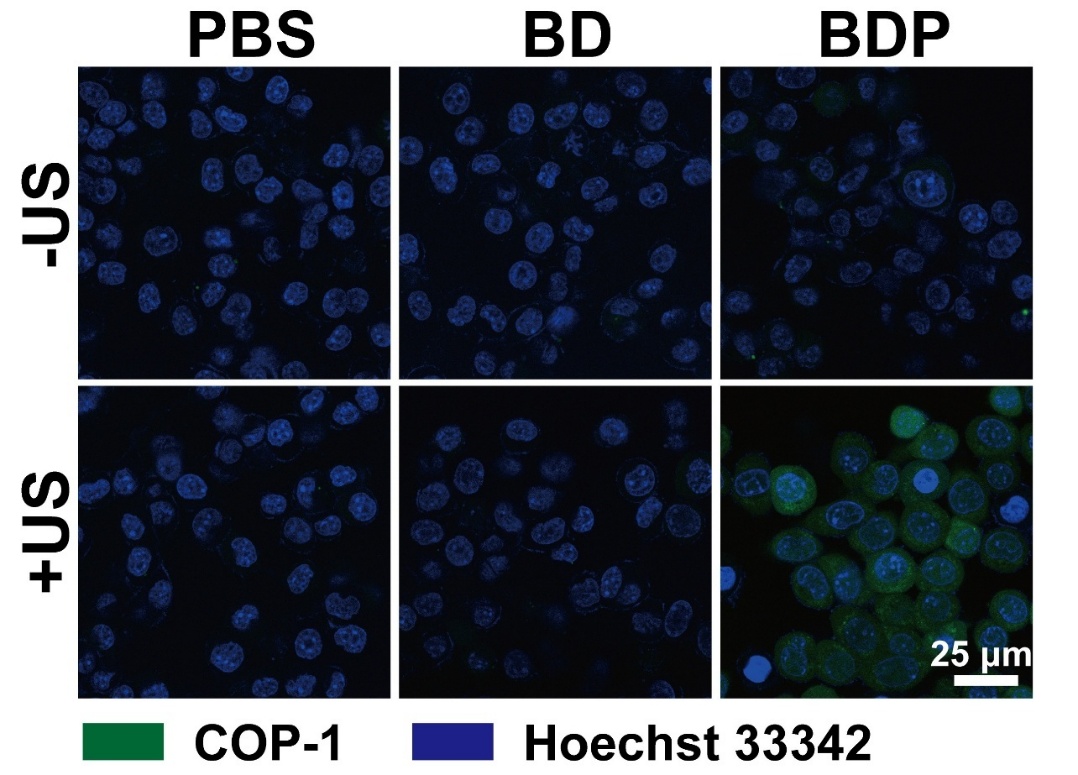


**Figure S30.** CLSM images of CO in 4T1 cells treated with BD or BDP with or without US irradiation.


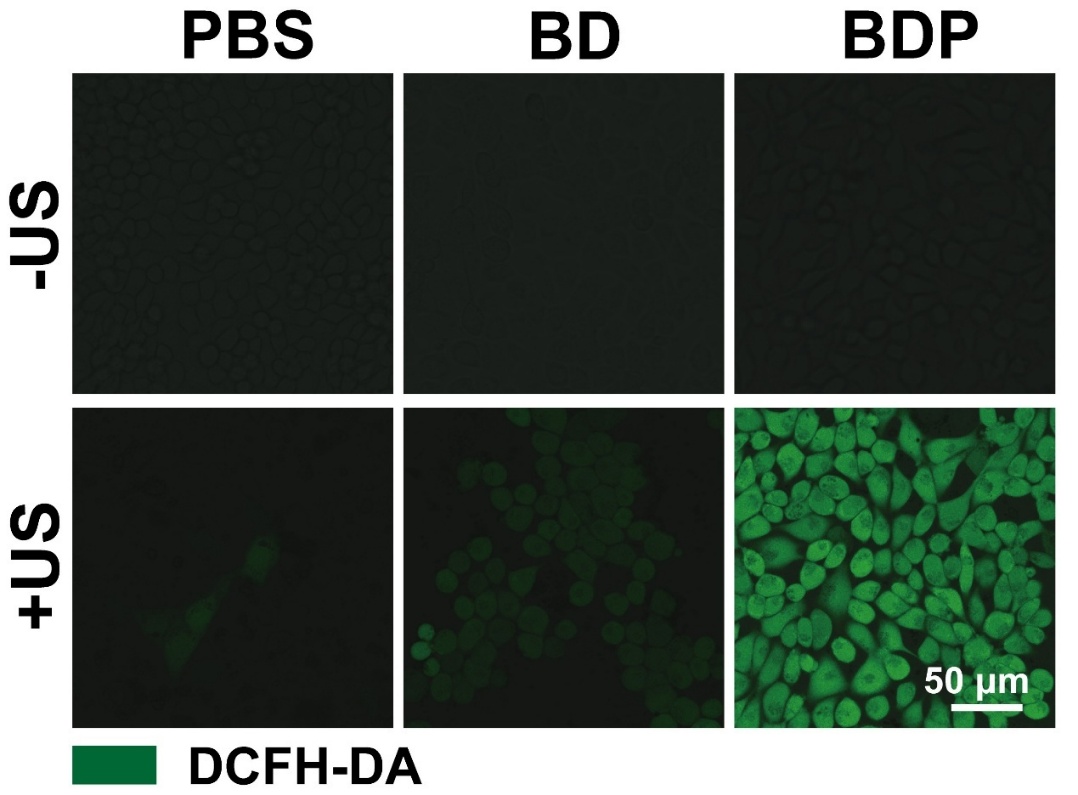


**Figure S31**. CLSM images of ROS (green: DCFH-DA staining) in 4T1 cells after different treatments.


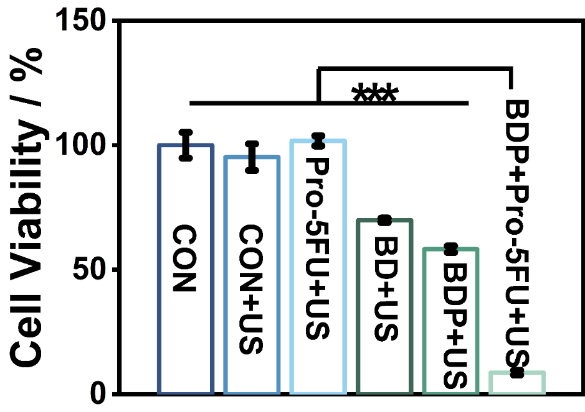


**Figure S32.** Cell viability of 4T1 cells after different treatments, including CON, CON+US, Pro-FU+US, BD+US, BDP+US, and BDP+Pro-5FU+US. Data were presented as mean ± SD (n = 5). Significance was calculated using one-way ANOVA and Tukey’s multiple comparisons test. *P < 0.05, **P < 0.01, ***P < 0.001, n.s. represents no significant difference.


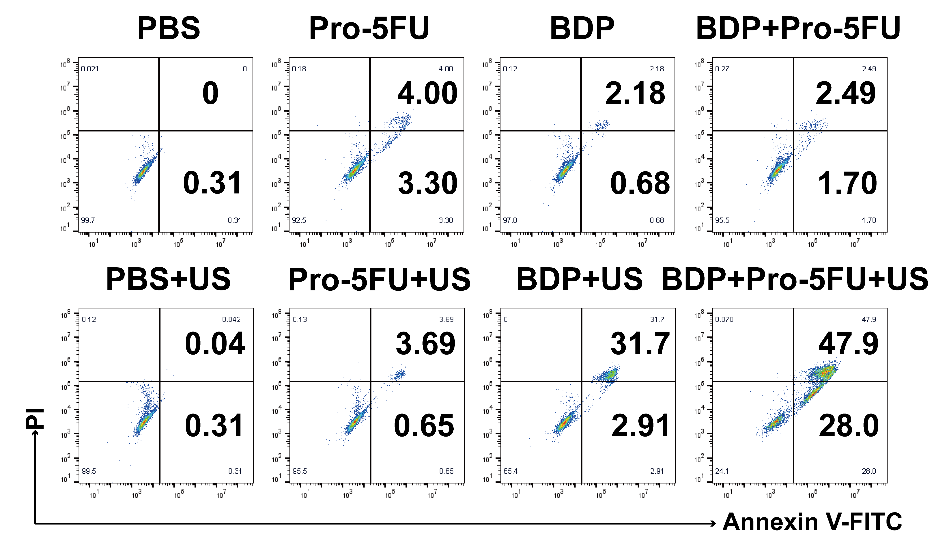


**Figure S33**. Flow-cytometry-based apoptosis assay using Annexin V/PI staining.


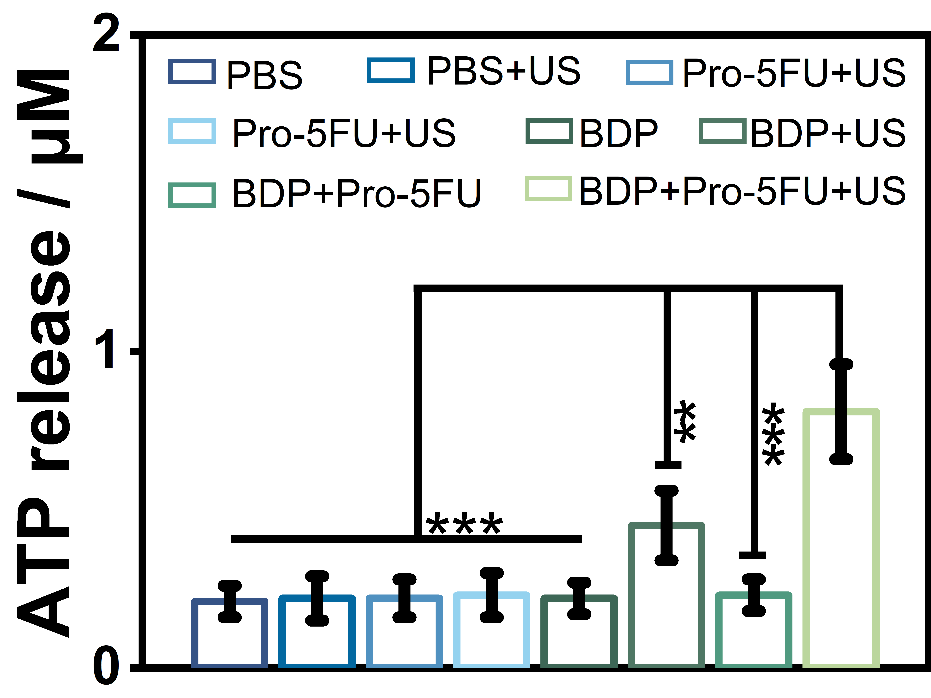


**Figure S34**. ATP secretion after the various treatments. Data were presented as mean ± SD (n = 5). Significance was calculated using one-way ANOVA and Tukey’s multiple comparisons test. *P < 0.05, **P < 0.01, ***P < 0.001, n.s. represents no significant difference.


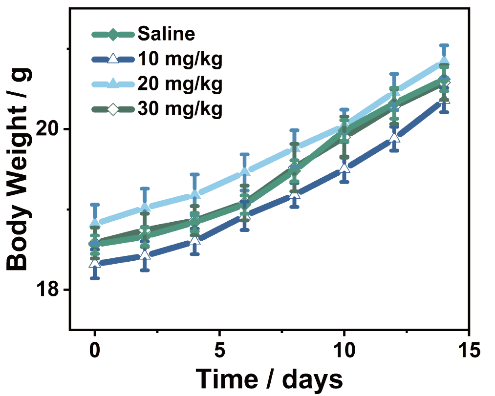


**Figure S35**. Curves of body weight change in mice after treatment with different dosages of BDP. Data were presented as mean ± SD (n = 5).


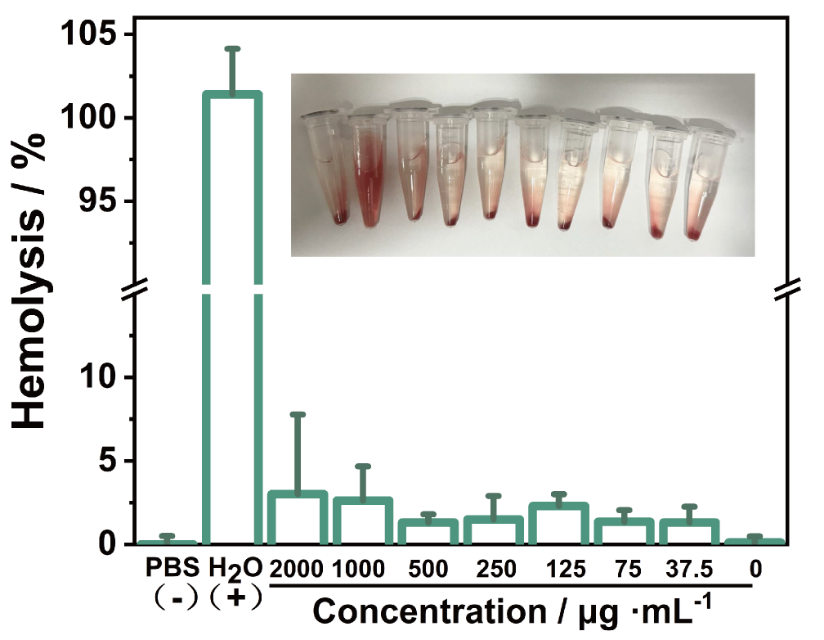


**Figure S36**. Hemolytic effects of different concentrations of BDP. Data were presented as mean ± SD (n = 5).


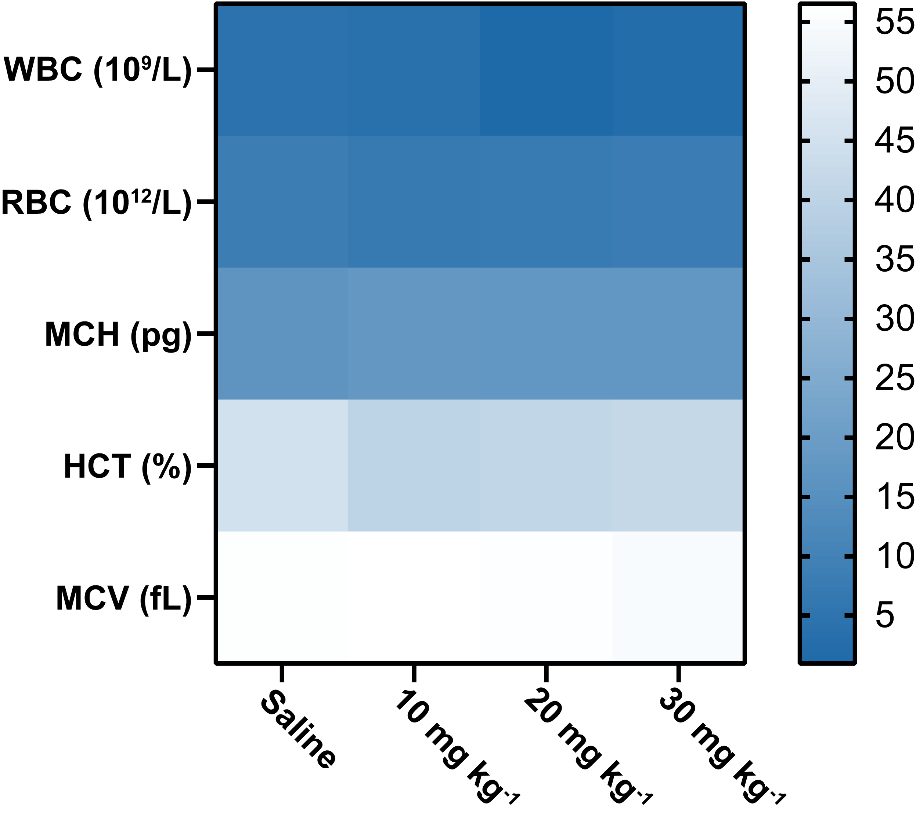


**Figure S37**. Blood routine analysis of white blood cells (WBC), red blood cells (RBC), mean corpuscular hemoglobin (MCH), hematocrit (HCT), and mean corpuscular volume (MCV). n=3.


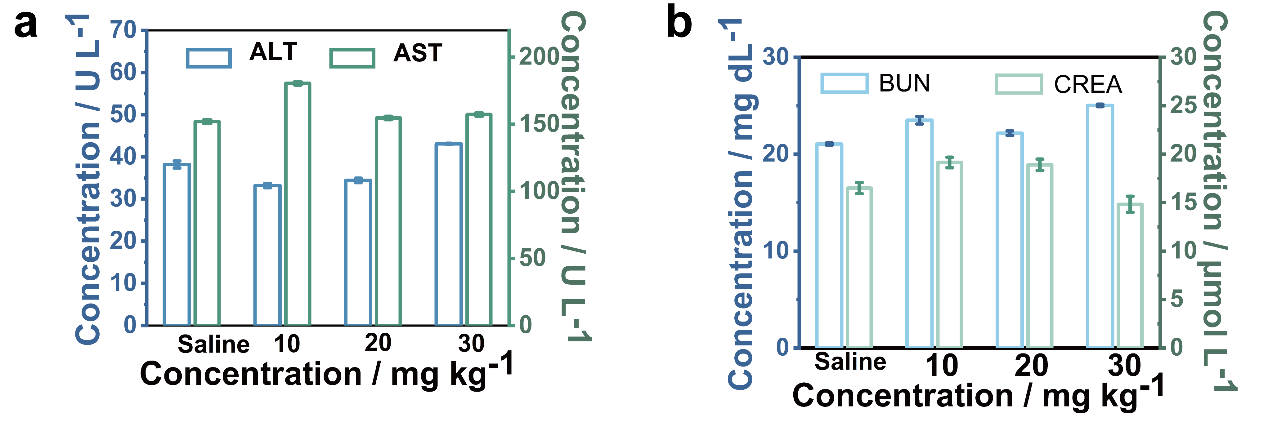


**Figure S38**. Biochemical analysis of liver function (a), including alanine aminotransferase (ALT), aspartate aminotransferase (AST), as well as kidney function (b), including creatinine (CREA), and Blood Urea Nitrogen (BUN). Data were presented as mean ± SD (n = 3).


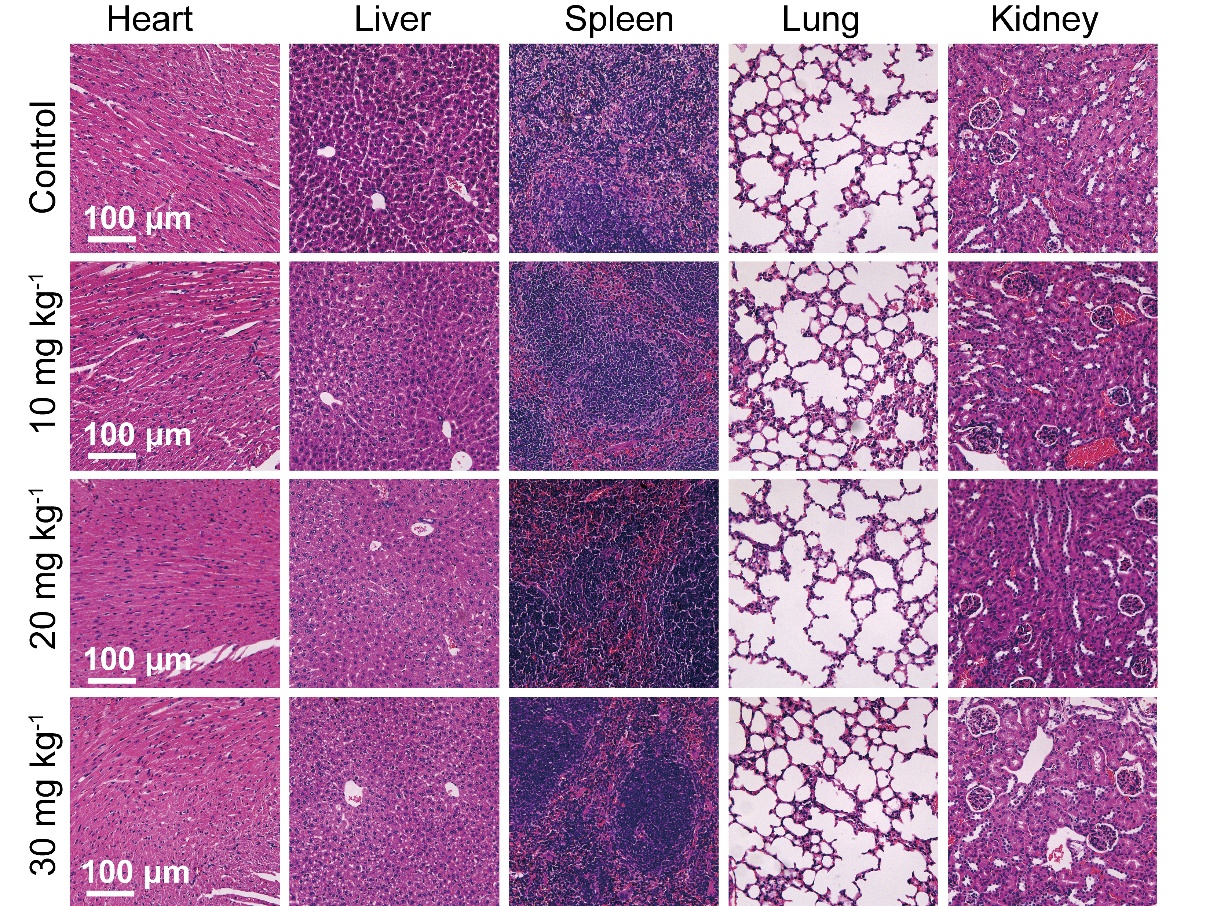


**Figure S39**. Histological images of H&E-stained major organ slices.


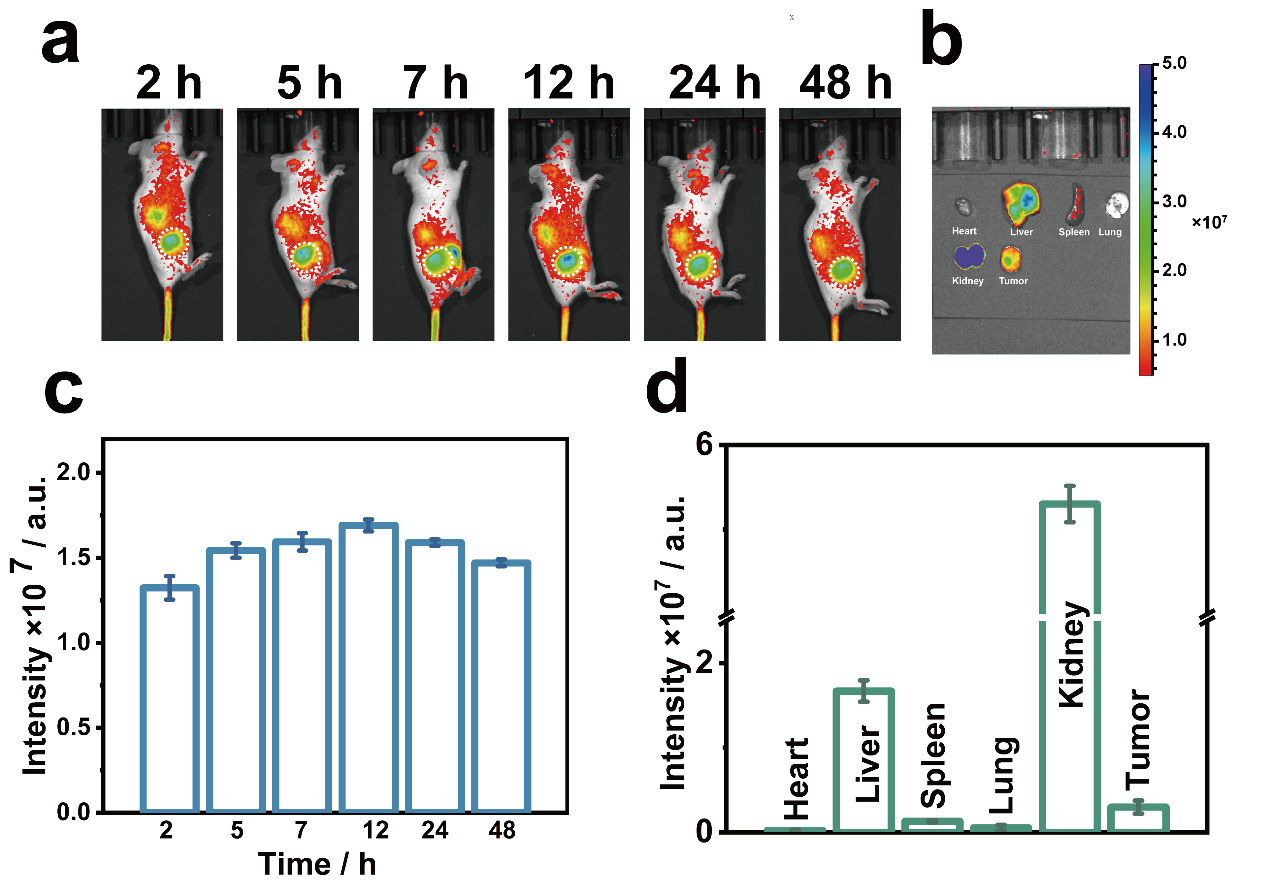


**Figure S40**. (a) *In vivo* fluorescence images of tumor regions at different time points after tail vein injection of Cy7-labeled BDP. (b) Ex vivo fluorescence images of major organs and tumors dissected from mice at 48 h post-injection. (c) Fluorescence statistics of tumor fluorescence after different times *of* injection. (d) Fluorescence statistics of major organs and tumors dissected from mice at 48 h post-injection. Data were presented as mean ± SD (n = 3).


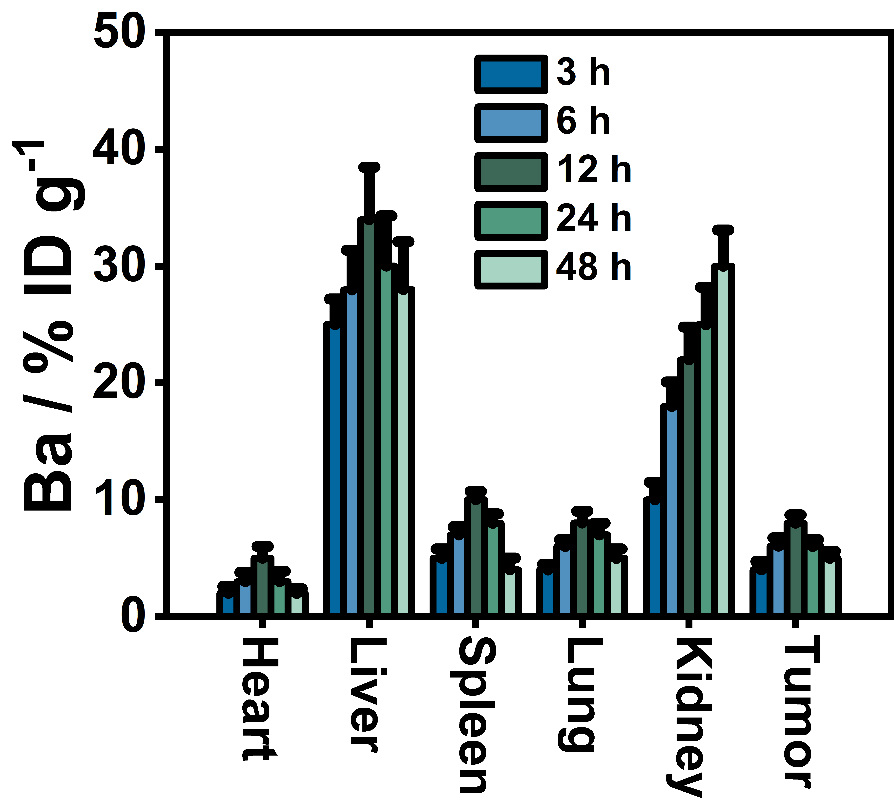


**Figure S41.** Biodistribution of BDP particles at 3, 6, 12, 24, and 48 hours following tail vein injection. Data were presented as mean ± SD (n = 3).


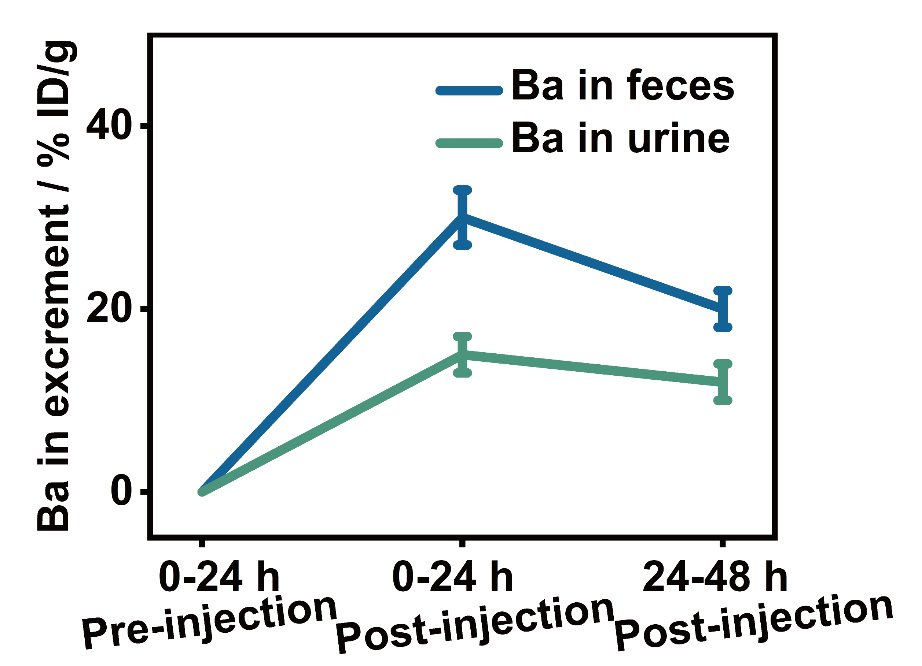


**Figure S42**. Barium content in feces and urine at different time points after tail vein injection of BDP. Data were presented as mean ± SD (n = 5).


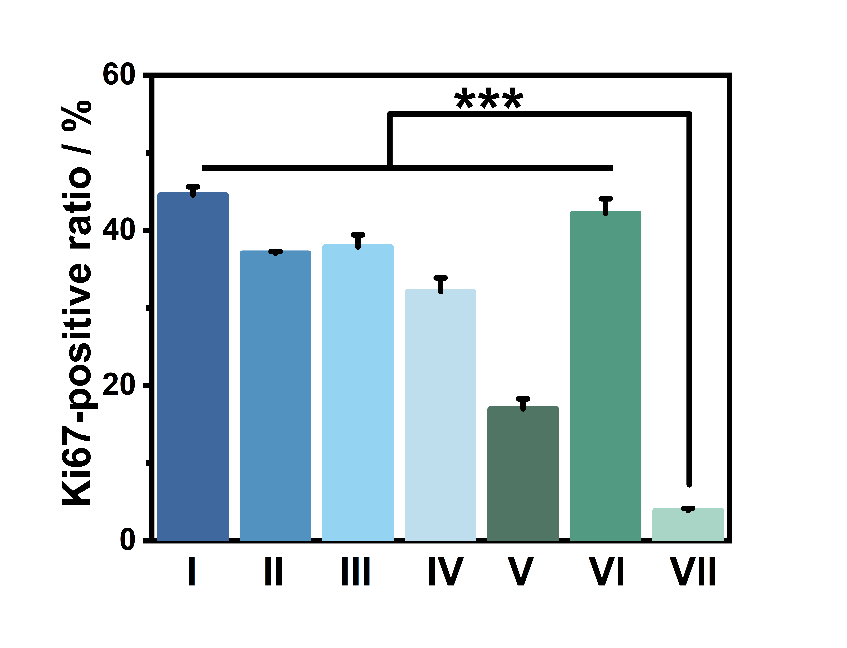


**Figure S43**. Quantification of Ki67-positive cells. Data were presented as mean ± SD (n = 3). Significance was calculated using one-way ANOVA and Tukey’s multiple comparisons test. *P < 0.05, **P < 0.01, ***P < 0.001, n.s. represents no significant difference.





**Figure S44**. Quantification of TUNEL-positive cells. Data were presented as mean ± SD (n = 3). Significance was calculated using one-way ANOVA and Tukey’s multiple comparisons test. *P < 0.05, **P < 0.01, ***P < 0.001, n.s. represents no significant difference.


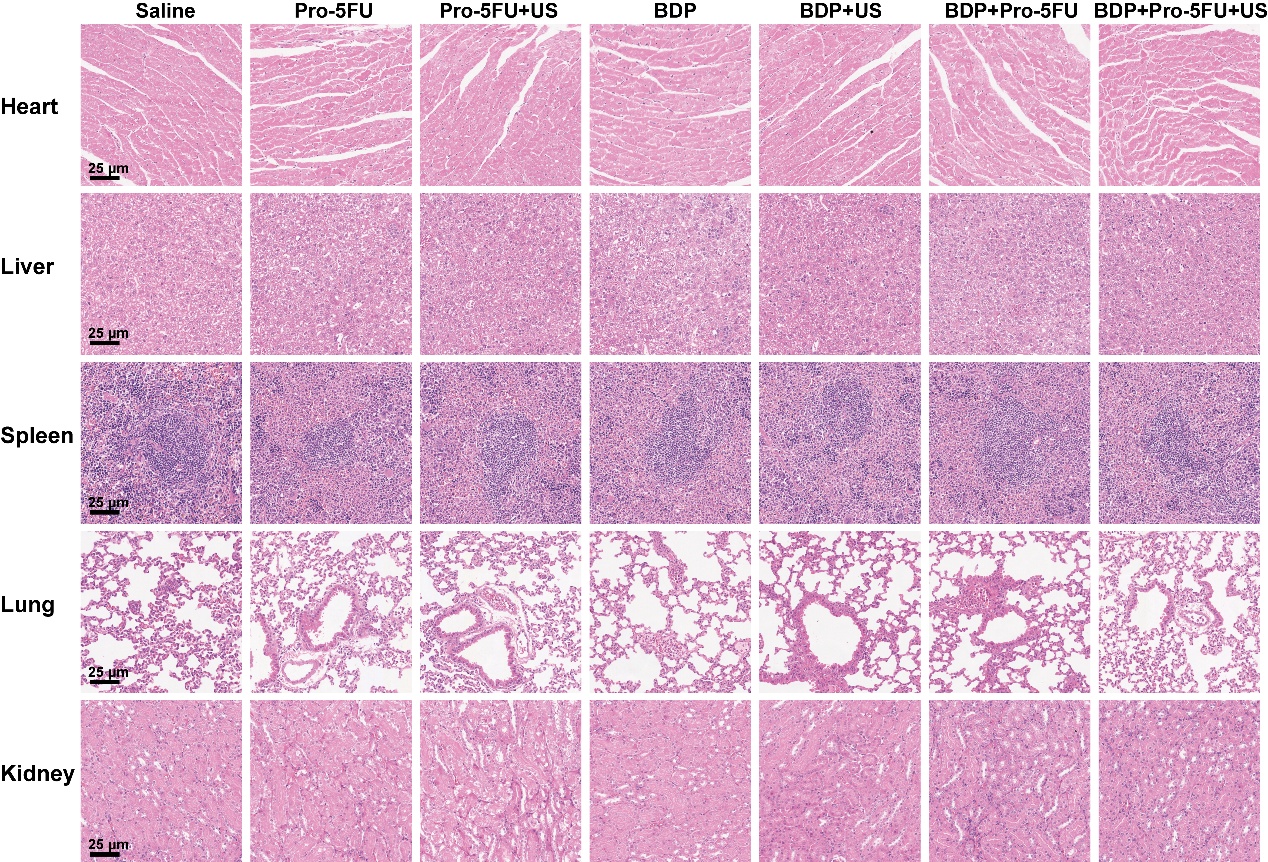


**Figure S45**. Representative histological images of H&E-stained major organ slices after 14 days of treatment.


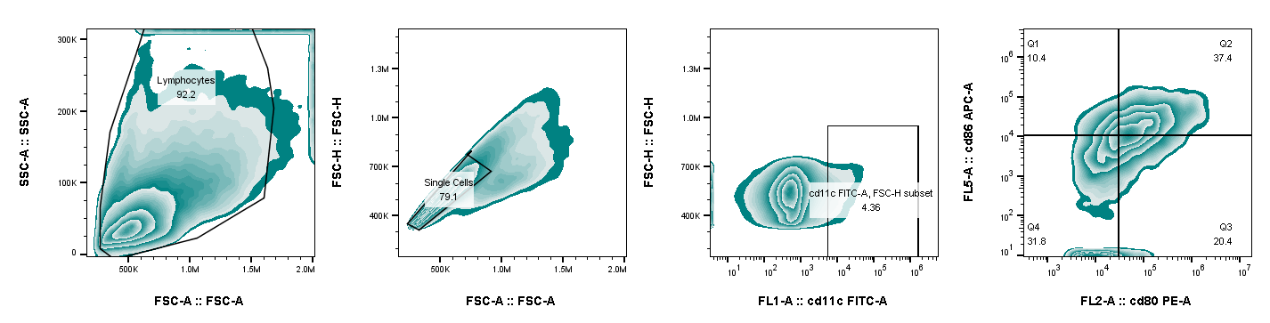


**Figure S46**. Gating strategies of the flow cytometry for DC cells in TDLNs.


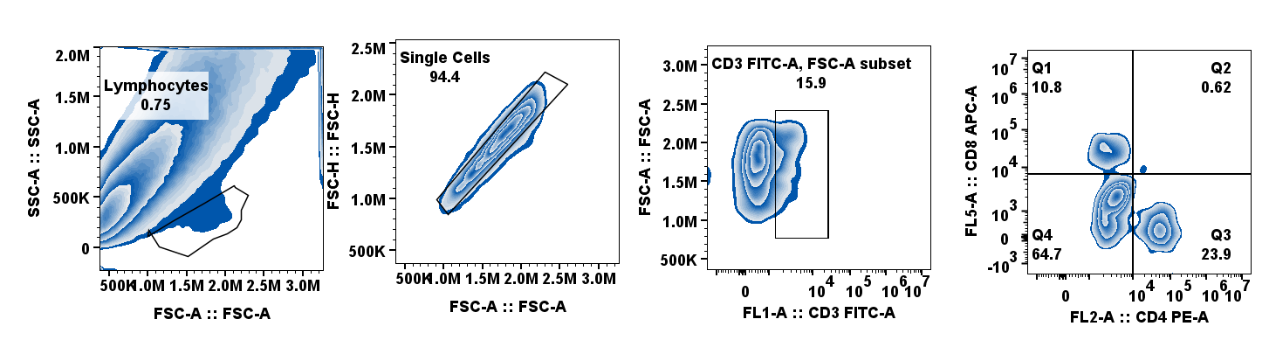


**Figure S47**. Gating strategies of the flow cytometry for CD3^+^CD4^+^ and CD3^+^CD8^+^ T cells in the tumors.


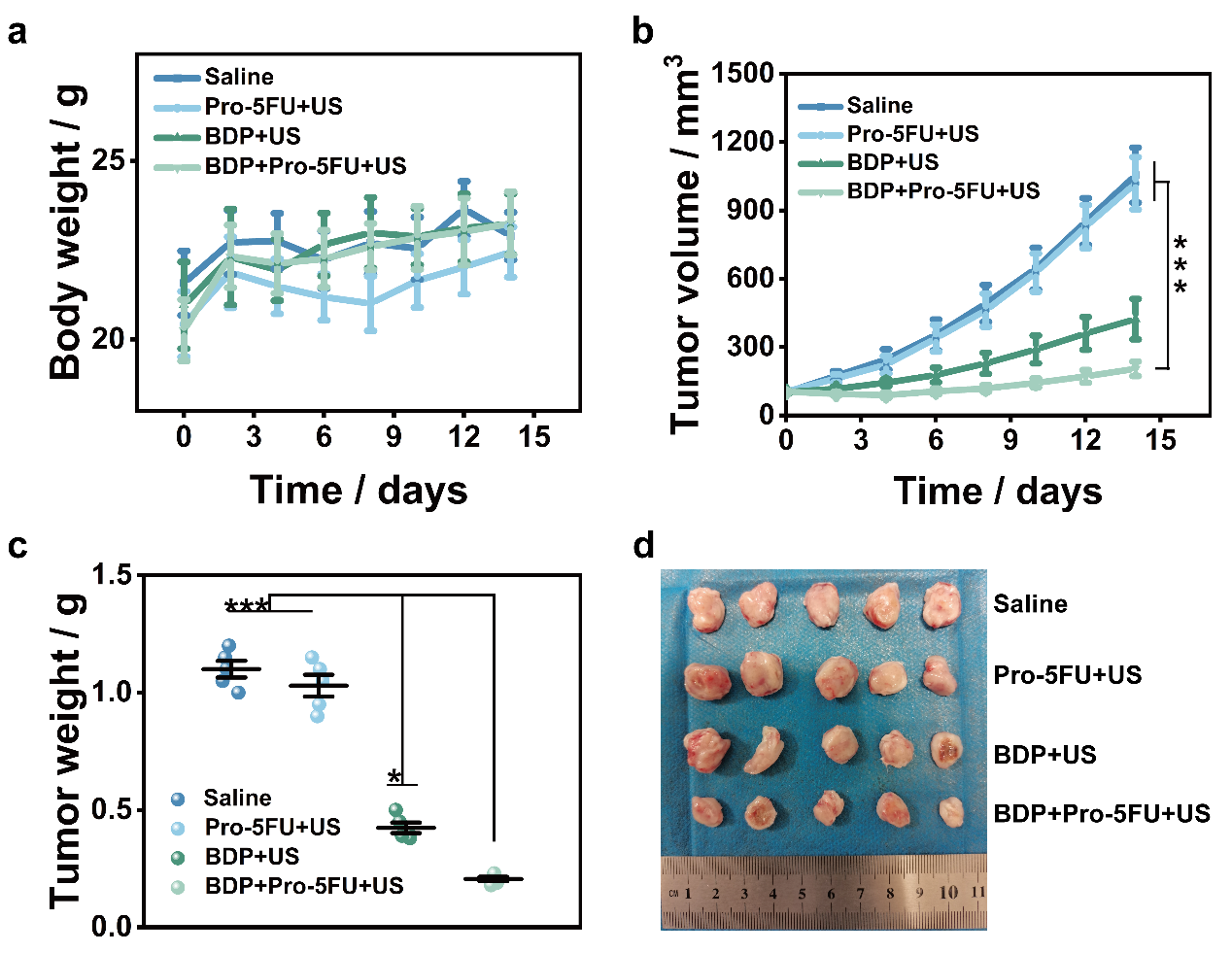


**Figure S48.** Evaluation of the in vivo antitumor efficacy of the prodrug via tail vein administration using piezocatalytic bioorthogonal chemistry. (a) Body weight changes of tumor-bearing mice during treatments. Data were presented as mean ± SD (n=5). (b) Relative tumor volume from 0 to 14 days during treatments. Data were presented as mean ± SD (n=5). (c) Tumor weight of mice after treatments. Data were presented as mean ± SD (n=5). (d) Photographs of ex vivo tumors obtained on the 14th day. Significance was calculated using one-way ANOVA and Tukey’s multiple comparisons test. *P < 0.05, **P < 0.01, ***P < 0.001, n.s. represents no significant difference.

| Materials | Pro-drug | Concentration | Activate method | Cell line | Cell viability | Reference |
| --- | --- | --- | --- | --- | --- | --- |
| Pd^0^ | Pro-5FU | 100 μM | Always on | HCT116 | ~5% | [1] |
| Pd^0^ | Pro-5FU | 30 μM | Always on | BxPC-3 | ~10% | [1] |
| Pd/BP | Pro-5FU | 500 μM | Always on | Hela | ~21% | [2] |
| LM-Pd | Pro-5FU | 1 mM | Always on | CT26 | ~30% | [3] |
| LM-Pd | Pro-5FU | 1 mM | Always on | A549 | ~40% | [3] |
| Lipo-Pd@Au | Pro-5FU | 100 μM | NIR | HCT116 | ~10% | [4] |
| BDP | Pro-5FU | 500 μM | US | 4T1 | ~5% | Our Strategy |

**Table S1.** Comparison of recent prodrug activation and the activation scheme in cells.

| Group | Pro-drug | Concentration | Time | Conversion |
| --- | --- | --- | --- | --- |
| BDP+US | Pro-5FU | 500 μM | 5 min | 12.8% |
| BDP+US | Pro-5FU | 500 μM | 1 h | 51.2% |
| BDP+US | Pro-5FU | 500 μM | 2 h | 64.1% |
| BDP+US | Pro-5FU | 500 μM | 3 h | 80.8% |
| BDP+US | Pro-5FU | 500 μM | 4 h | 99.7% |
|  |  |  |  |  |

**Table S2.** Conversion of prodrug Pro-5FU in cells by 200 μg mL^-1^ BDP under different ultrasound exposure times (1.0 MHz, 1.0 W cm^-2^, 50% duty cycle).

**Reference:**

[1] Weiss, J. T.; Dawson, J. C.; Macleod, K. G.; Rybski, W.; Fraser, C.; Torres-Sánchez, C.; Patton, E. E.; Bradley, M.; Carragher, N. O.; Unciti-Broceta, A. Extracellular palladium-catalysed dealkylation of 5-fluoro-1-propargyl-uracil as a bioorthogonally activated prodrug approach. *Nat. Commun.* **2014**, *5*, 3277.

[2] Rong, M.; Liu, J.; Sun, Z.; Li, T.; Li, Y.; Jiang, C.; Lu, L. Rational Utilization of Black Phosphorus Nanosheets to Enhance Palladium-Mediated Bioorthogonal Catalytic Activity for Activation of Therapeutics. *Angewandte Chemie International Edition* **2023**, *62*, e202216822.

[3] Zhang, L.; Sang, Y.; Liu, Z.; Wang, W.; Liu, Z.; Deng, Q.; You, Y.; Ren, J.; Qu, X. Liquid Metal as Bioinspired and Unusual Modulator in Bioorthogonal Catalysis for Tumor Inhibition Therapy. *Angewandte Chemie International Edition* **2023**, *62*, e202218159.

[4] Ortega-Liebana, M. C.; Travnickova, J.; Adam, C.; González-Calderón, D.; Lorente-Macías, Á.; Lochenie, C.; Arenal, R.; Patton, E. E.; Unciti-Broceta, A. Near-Infrared Light-Accelerated Bioorthogonal Drug Uncaging and Photothermal Ablation by Anisotropic Pd@Au Plasmonic Nanorods. *J. Am. Chem. Soc.* **2025**, *147*, 23980-23990.
